# Representing Context in FrameNet: A Multidimensional, Multimodal Approach

**DOI:** 10.3389/fpsyg.2022.838441

**Published:** 2022-04-04

**Authors:** Tiago Timponi Torrent, Ely Edison da Silva Matos, Frederico Belcavello, Marcelo Viridiano, Maucha Andrade Gamonal, Alexandre Diniz da Costa, Mateus Coutinho Marim

**Affiliations:** ^1^FrameNet Brasil, Graduate Program in Linguistics, Faculty of Letters, Federal University of Juiz de Fora, Juiz de Fora, Brazil; ^2^Laboratório Experimental de Tradução, Graduate Program in Linguistics, Faculty of Letters, Federal University of Minas Gerais, Belo Horizonte, Brazil

**Keywords:** FrameNet, qualia structure, multimodal semantic representation, domain adaptation in neural machine translation, context

## Abstract

Frame Semantics includes context as a central aspect of the theory. Frames themselves can be regarded as a representation of the immediate context against which meaning is to be construed. Moreover, the notion of frame invocation includes context as one possible source of information comprehenders use to construe meaning. As the original implementation of Frame Semantics, Berkeley FrameNet is capable of providing computational representations of some aspects of context, but not all of them. In this article, we present FrameNet Brasil: a framenet enriched with qualia relations and capable of taking other semiotic modes as input data, namely pictures and videos. We claim that such an enriched model is capable of addressing other types of contextual information in a framenet, namely sentence-level cotext and commonsense knowledge. We demonstrate how the FrameNet Brasil software infrastructure addresses contextual information in both database construction and corpora annotation. We present the guidelines for the construction of two multimodal datasets whose annotations represent contextual information and also report on two experiments: (i) the identification of frame-evoking lexical units in sentences and (ii) a methodology for domain adaptation in Neural Machine Translation that leverages frames and qualia for representing sentence-level context. Experimental results emphasize the importance of computationally representing contextual information in a principled structured fashion as opposed to trying to derive it from the manipulation of linguistic form alone.

## 1. Introduction

Recent advances in Computational Linguistics derived from the use of contextualized embeddings such as BERT (Devlin et al., 2019) and ELMo (Peters et al., 2018) have brought contextual information to the core of Natural Language Processing (NLP), raising key issues on which kinds of information are captured by and on how they are represented in those models (Rogers et al., 2020; Bender et al., 2021; Bommasani et al., 2021). Context, in this scenario, is broadly understood as information extracted from the set of words occurring around a given word token (Smith, 2020; Xia et al., 2020). The term context is also used in NLP to refer to information that can be extracted from sentences around the one being analyzed for a given task, such as Natural Language Generation (NLG) for question answering and dialogue systems (Zhou et al., 2016) or context-aware semantic parsing (Li et al., 2020b). Also, identifying and modeling commonsense knowledge is a key aspect of tasks involving Natural Language Understanding (NLU) and Inference (NLI) (LoBue and Yates, 2011; Sap et al., 2020).

There is no consensus in linguistic theory as to a definition of context. As didactically explained by Schifrrin (1994), theories may see context as the commonsense knowledge needed to engage in and maintain communication, the situational framing, the text surrounding the phenomena under analysis, or a combination of two or three of them. Frame Semantics (Fillmore, 1982) includes all three aspects of context listed by Schifrrin (1994) to some extent. As one of the *Semantics of Understanding*, Frame Semantics aims “*to uncover the nature of the relationship between linguistic texts and the interpreter's full understanding of texts in their contexts”* (Fillmore, 1985, p.231).

Frames are defined as the background scenes against which the meaning of some linguistic material should be construed. Such scenes are composed of participants and props, each of which is defined as a concept or Frame Element (FE). FEs are related in such a way that the presence of one of them brings the others into play (Fillmore, 1982, p.111). Frames can thus be regarded as capable of representing important elements of commonsense knowledge required for properly understanding the meaning of a given piece of language. Moreover, when defining the theory, Fillmore recognized that another important type of framing was the *framing of the actual communication situation* (Fillmore, 1982, p.117), highlighting the centrality of pragmatic frames for Frame Semantics [see Czulo et al. (2020) for discussion]. Finally, the surrounding text is the starting point of any frame semantic analysis, since frames are instantiated either via their evocation by specific linguistic units, or via their invocation by comprehenders from the combination of clues found in text.

Frame Semantics has been computationally implemented in the form of framenets, first for English (Baker et al., 1998), and then for several other languages, including Brazilian Portuguese, by FrameNet Brasil (Torrent and Ellsworth, 2013). Because they are structured according to the principles of Frame Semantics, framenets are in theory capable of computationally representing diverse aspects of context. In this article, we discuss the extent to which framenets are capable of representing context and demonstrate that enriching the original framenet database structure with other dimensions of meaning representation and other communicative modes augments this capacity.

To achieve this goal, we start by briefly presenting the original Berkeley FrameNet model in section 2 and the dimensions of context it captures. Next, in section 3, we present three new relations added to FrameNet Brasil (FN-Br) and discuss how they add new dimensions of meaning representation to the framenet model. Section 4 demonstrates how the FN-Br model can be extended to represent contextual information captured by the analysis of other communication modes, such as video, for example. In section 5, two experiments based on the proposals presented in the previous two sections have their results reported on, while section 6 presents the conclusions of this article.

## 2. The Original FrameNet Structure

**Figure 1:**
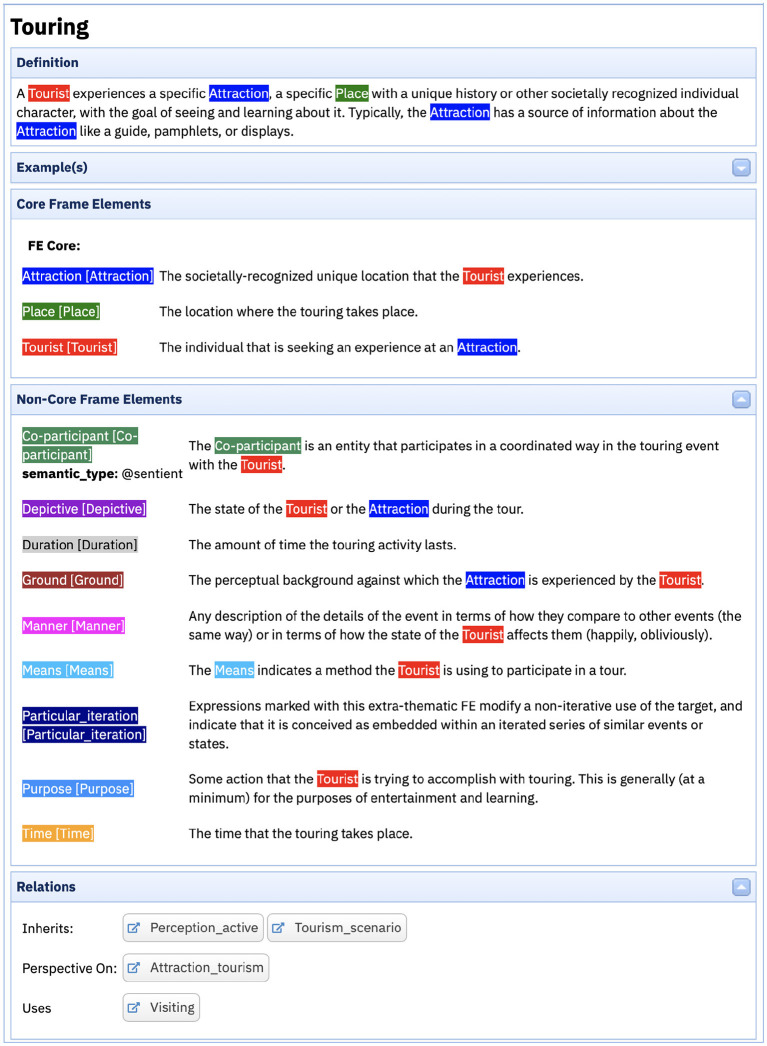
The Touring frame.

Berkeley FrameNet (BFN) (Baker et al., 1998; Fillmore and Baker, 2009) is the original implementation of Frame Semantics. It started as a lexicon of the English language (Fillmore et al., 2003), which was then expanded to include other types of linguistic structure, namely constructions (Fillmore et al., 2012). Each lexical unity (LU) in BFN is the pairing of a lemma and a frame representing the background context against which the LU definition is to be understood. Consider, for example, the Touring frame in Figure 1, evoked by LUs such as *see.v, tour.v, tour.n, visit.v* among others.

Each frame comprises a definition and a set of Frame Elements (FEs). The definition provides a general description of the scene represented by the frame and, in most cases, it references the FEs. FEs, in turn, are classified into core and non-core, the first being mandatory for the instantiation of the frame, and the latter indicating circumstantial information that may appear in sentences featuring the frame-evoking LU (Ruppenhofer et al., 2016). In the Touring frame, the core FEs are Attraction, Place and Tourist. And, as non-core, there are some like Co-participant, which is an entity that participates in a coordinated way in the touring event with the Tourist, and Time, which identifies when the touring activity takes place.

**Figure 2:**
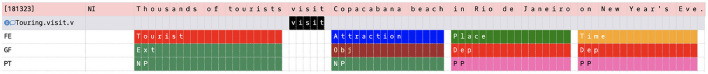
Example of a FrameNet Annotation Set.

FrameNet annotation consists of syntactically and semantically analyzing a target LU. The multi-layer lexicographic annotation consists of at least three layers of annotation: one for Frame Elements (FE), one for Grammatical Functions (GF), and another for Phrase Types (PT). Figure 2 provides an example of a framenet annotation set for the LU *visit.v* evoking the Touring frame. Note that the annotation assigns both semantic and morphosyntactic metadata to the sentential context—also referred to as cotext–of the target LU.

Fundamentally, frames are not a set of isolated units. They are associated with other frames via a set of typed relations, or, in other words, frames are organized in a net-like structure. It is important to note that every frame-to-frame relation is sustained by relations holding between the FEs in each frame. BFN defines seven types of relations together with a meta-relation named *See_also*, used for editorial purposes. Fillmore and Baker (2009) classify those relations in three groups: generalization, event structure and systematic relations.

- **Generalization Relations**
  - **Inheritance**: Similarly to the idea of subsumption, common in ontologies and knowledge graphs, this relation indicates that all facts that are strictly true for the semantics of the mother frame must correspond to some equally or more specific facts associated with the daughter frame. The FEs in the mother frame must be associated with those in the daughter frame, but the latter may have different names and definitions. Also, the daughter frame may have more FEs than the mother frame. Multiple inheritance is allowed.
  - **Perspective_on**: This relation implements the idea of profiling, taking the Figure-Ground distinction into consideration. Different lexical items—for example, *buy.v* and *sell.v*—may refer to an event of goods transfer, but they do so from two different perspectives, represented by the Commerce_buy and the Commerce_sell frames. This allows for the profiling of different aspects relevant to the context.
  - **Using**: This relation is mostly used for cases where part of the scene in the daughter frame refers to the mother frame. Fillmore and Baker (2009) explain that the daughter frame depends on the background knowledge of the mother frame, meaning that at least some of the core FEs in both of them should be related.
- **Event Structure Relations**
  - **Subframe**: This relation represents the possibility of a merological interpretation for events, that is, the daughter frame is expressed as a sub-event of a more complex mother event.
  - **Precedes**: This relation indicates that there is a temporal order between frames—the mother frame precedes the daughter frame—allowing for some basic inference about preceding and following events.
- **Systematic Relations**
  - **Causative_of**: The mother frame represents a version of the daughter frame where the agent or cause of the event represented by the frame is profiled. This relation allows for inferring some systematic cause-effect processes.
  - **Inchoative_of**: Also related to cause-effect processes, this relation indicates that the mother frame depicts a change of state scene whose result is the daughter frame.

Taking the Touring frame as an example, it inherits from both Tourism_scenario and Perception_Active. In other words, it carries all the information of the parent frames in a more specific manner. That said, the Perceiver_agentive FE in the latter frame is specified as the Tourist FE in Touring, for example. Moreover, Touring is a perspective on Attraction_tourism, which means that the first adopts the point of view of the tourist, while the latter is neutral to perspectivization. It also uses the Visiting frame, that is, to understand Touring, we need to consider the idea of Visiting in the background.

Because BFN combines a structured database with annotation associating the categories in this database to linguistic data, contextual information can be represented in BFN by the frames, FEs and relations between them, by the annotated sentences, or by a combination of both. In a nutshell, the original BFN structure can capture contextual information of the following types:

- Commonsense knowledge about the participants and props usually involved in a given type of event, state or relation, as well as information on their nature and on attributes that can be assigned to them. This information is represented, from the structural point of view, via FEs and their statuses in the frame. From the annotated data perspective, it is represented by the valence descriptions extracted from the annotation.
- Commonsense knowledge about the events, states, relations, attributes and entities related to the main predicate in a given sentence. This information is structurally represented via frame-to-frame relations. It can also be represented in full-text annotation, when all lexical units are treated as targets and their dependents are annotated.

Although those types of contextual information are highly relevant, the BFN structure is not capable of representing other important aspects of context already recognized as central in Frame Semantics theory (Fillmore, 1982, 1985). Regarding the contextual information anchored in the text surrounding the LU under analysis—or cotext—, the BFN model can only partially capture them via annotation. Returning to the example in Figure 2, note that the Attraction FE is assigned to the NP *Copacabana beach*. Hence, in this piece of annotation, we could associate the head of the NP *beach.n* with the concept of Attraction. This association represents a piece of commonsense knowledge about tourism, namely that natural features such as beaches, mountains and lakes have the potential to become tourist attractions. However, the current BFN structure has no means of storing the generalization expressed in the previous sentence. To properly represent this and other types of contextual information in FrameNet, we propose adding other dimensions of meaning to the current frame structure. Such a proposal is detailed next.

## 3. A Multidimensional FrameNet: Enriching Frame Structure

As described in section 2, the BFN structure is not capable of representing all contextual information associated with commonsense knowledge whose importance to meaning construction is recognized in Frame Semantics. In this section, we discuss the extent to which three additions to the FrameNet database structure proposed by FrameNet Brasil (Torrent et al., submitted^1^)—FE-to-frame, metonymy and ternary qualia relations—can enhance the representation of context.

### 3.1. The Frame Element to Frame Relation

The FE-to-frame relation models the fact that a given FE in a frame may reference another frame in a framenet. Such mapping does not necessarily apply to the definition of a semantic type to the FE. Its purpose is to extend the conceptual interpretation of FEs so that, besides representing the instantiation of micro-thematic functions in an annotation set, they may also represent commonsense knowledge.

Let us return to the example of the Touring frame in Figure 1. This frame features three core FEs: Tourist, Attraction, and Place. As the discussion of the annotation set in Figure 2 reveals, it is common that a part of the sentence instantiating an FE contains a lexical item that may evoke another frame. In the case of the sentence in Figure 2, this is what happens with the Tourist FE, which is instantiated by the noun phrase *thousands of tourists*. The noun *tourist* in this noun phrase evokes the People_by_leisure_activity frame. One alternative for representing those kinds of connections between frames in the database could be proposing a Using relation between People_by_leisure_activity and Touring. Nonetheless, such a solution would not be capable of identifying that the part of the latter that refers to the first is the Tourist FE.

**Figure 3:**
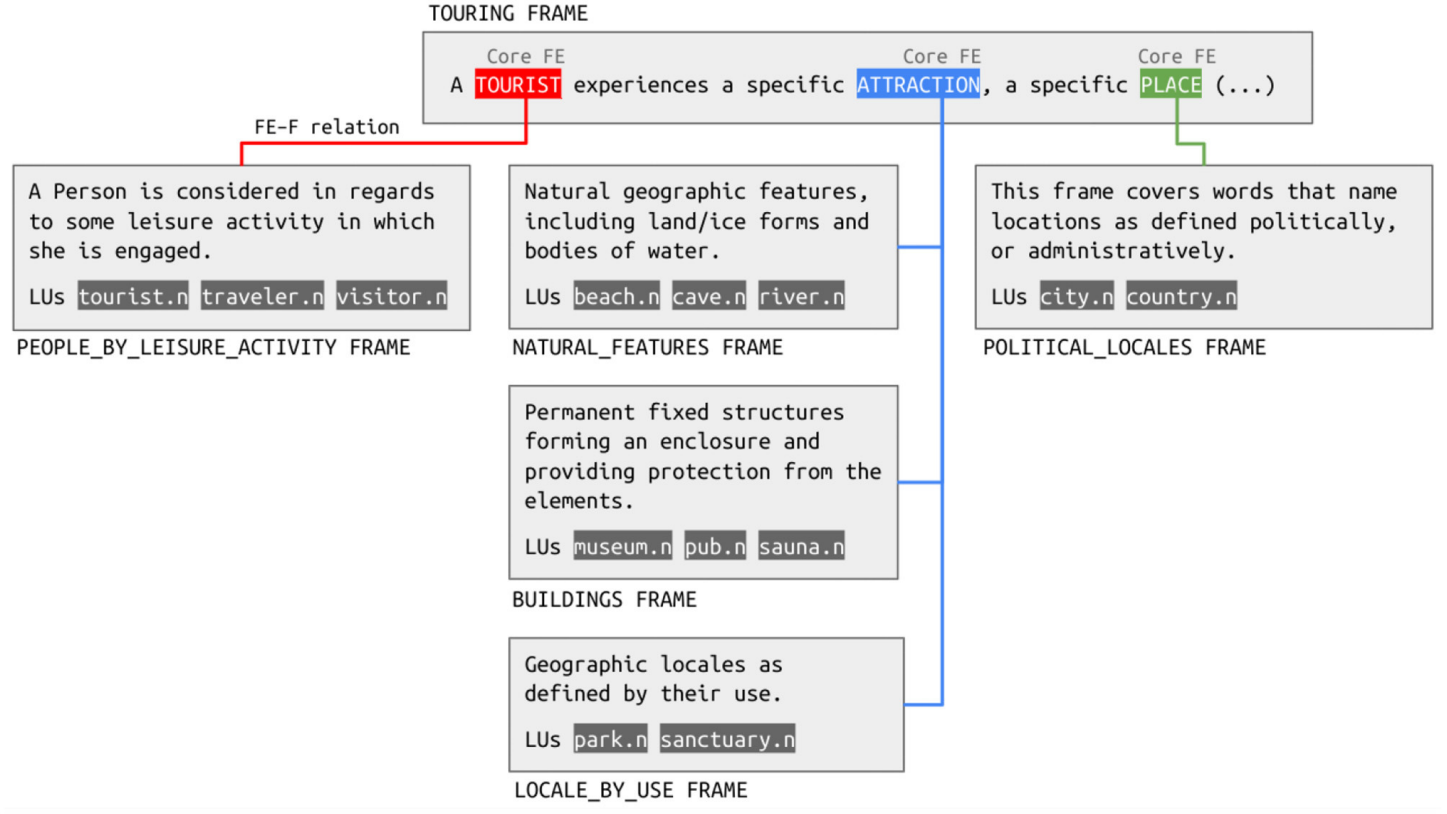
Frame Element-to-Frame relations in the Touring frame.

To properly represent which concept in the system of concepts—the frame—is linked to another frame, FN-Br created the FE-to-frame relation. In the Touring frame, this relation maps not only the Tourist FE to the People_by_leisure_activity frame, but also the Place FE to the Locale frame and the Attraction FE to the Natural_features, Buildings and Locale_by_use frames, as shown in Figure 3.

The instances of the FE-to-frame relation depicted in Figure 3 are good examples of the policies for creating this relation devised by FN-Br. There are two eligibility criteria used to decide whether a given FE in a given frame is a candidate for the relation. First, it is important to note that frames representing entities are not eligible for the FE-to-frame relation. This policy has the purpose of avoiding the creation of loops in the frame graph. A frame such as People, for example, features the FE Person. If we were to assign an FE-to-frame relation for the Person FE, it would be related to the People frame, creating a loop. Second, the FE-to-frame relation only applies to core FEs, not to non-core ones, since, as pointed out by Torrent et al. (2018), non-core FEs are sometimes proposed in BFN to account for phenomena that would be more properly addressed in a Constructicon, that is, a repository of grammatical constructions. The Touring frame meets both eligibility criteria, and, therefore, its core FEs are candidates to the FE-to-frame relation.

Next, each FE is analyzed for the aspect of the scene they represent. From this analysis, a linguist may assign one or more frames to the FE. Linguists should choose the frame(s) most informative for modeling the semantics of each FE. Informativeness, in this case, translates into choosing the frame that is, at the same time, as general as possible, so that it can represent the semantics of the FE as broadly as possible, and as specific as possible, so that this representation does not include information that is not relevant for the FE. When positing the FE-to-frame relations, linguists followed the guidelines presented next:

1. For each frame, analyze the type of concept it models. Only frames indicating events, states, attributes and relations are eligible to The FE-to-frame relations. If the frame is an instance one of the four types mentioned, proceed to the next step. If not, restart the process with the next frame.
2. For each core FE in the selected frame, use information from the FE definition or semantic type to determine the type of concept it refers to.
3. For each concept type (e.g., people, place, event), search the most top-level frame representing it.
4. Analyze the inheritance chain of the top-level frame chosen, checking both the definitions of the frames in the chain and the LUs evoking them to choose the frame that represents the correct degree of granularity for the prototypical fillers of the FE for which the relation is being created.
5. Create the FE-to-frame relation.

Following those guidelines, the Tourist FE was mapped to the People_by_leisure_activity, not to the People frame, since the latter is too general for defining the semantics of Tourist. Similarly, the Place FE was mapped to the Political_locales frame and not to the Locale—too general—or to the Foreign_or_domestic_country—too specific—frames. The informativeness tension may also result in the proposition of more than one instance of the FE-to-frame relation to one same FE. This is the case of the Attraction FE, which is related to the Natural_features, Buildings and Locale_by_use frames. This is because those three frames do not have a single mother frame that is capable of properly representing the semantics of a tourist attraction. Their inheritance chain involves the Locale_by_ownership frame–evoked by LUs such as *estate.n* and *property*—and eventually reaches the Locale frame, which is also inherited by frames such as Locale_by_characteristic_entity—evoked by *enclave.n* and *quarter.n*—and Businesses—evoked by *appliance store.n* and *ATM.n*. As the LUs evoking those frames demonstrate, they should not be involved in the definition of what counts as a tourist attraction to the same extent that the LUs evoking the frames depicted in Figure 3 do.

The application of the eligibility criteria and the maximized informativeness principle to the FN-Br database resulted in the proposition of 3,582 instances of the FE-to-frame relation. Out of the 1,306 frames in FN-Br database, 1,198–91.7%–have at least one instance of the relation. The average of FE-to-frame relations per frame is 2.98 relations per frame. A total of 40 out of the 108 frames—37%—that do not feature any instance of the FE-to-frame relation represent entities. The remaining 63% are distributed between non-lexical and other very high-level frames representing abstract relations or image schemata. This is to say that virtually every frame in the FN-Br database representing an event, state, attribute or relation has at least one core FE linked to at least one other frame in the database. FE-to-frame relations represent a sensible increase in the granularity of semantic representation provided by FN-Br. For the sake of comparison, the FN-Br database features 1,846 frame-to-frame relations of the types defined in section 2, among those, 586 are Using relations. If we consider all relation types, FE-to-frame relations almost double the number of relations in the database. If we consider Using relations only, which would be the alternative to represent the mappings modeled by FE-to-frame relations, the latter increase by seven times the number of relations.

The FE-to-frame relation captures important aspects of the semantics of FEs, adding a new dimension of representation of contextual information, especially commonsense knowledge, to the framenet model. However, it is still not capable of modeling another pervasive phenomenon in language: that of metonymy. The next section addresses how this is accounted for in the FN-Br database.

### 3.2. Metonymic Substitution of Frame Elements

There is some consensus among researchers that both metaphor and metonymy are meaning mappings that can be differentiated by the nature of the source and target domains involved. While metaphor is a cross-domain or inter-domain mapping, meaning that at least two different domains are involved, metonymy is considered an intra-domain mapping, that is, it takes place within a single domain (Lakoff, 1979; Lakoff and Turner, 1989; Croft, 1993; Barcelona, 2002). The translation of such a distinction into the framenet domain results in the fact that metaphoric mappings are to be accounted for via frame-to-frame relations, while metonymic ones should be modeled as frame internal relations.

In the case of metaphor, the latest version of the Berkeley FrameNet *Book* (Ruppenhofer et al., 2016) includes guidelines on how metaphor should be addressed. Although no empirical validation of such a proposal has been reported yet, BFN already proposes a methodology for accounting for metaphoric mappings in the resource. In the case of metonymy, there are no theoretical-methodological proposals by the BFN team. Gamonal (2017) proposes a methodology for accounting for metonymic mappings in the FN-Br database via a relation between FEs, as it will be demonstrated next.

To exemplify the metonymic mapping, let us consider the sentence in (1), taken from the FN-Br corpus.

(1) São Paulo oferece bares, restaurantes, comida de rua, cantinas e pastelarias para praticamente todos os gostos. *São Paulo offers bars, restaurants, street food, cafeterias, and dumpling stores for almost all tastes*.

In (1), *São Paulo* is a metonymically named entity. According to Lakoff (1987, p.84), metonymies occur when concept A and concept B, which is closely associated to A, are both contained in a conceptual structure, and B is easier to understand, recognize or remember. Hence, referencing A via B is “more immediately useful for the given purpose in the given context.” In the example, we can interpret that there is a set of social organizations running businesses that offer food services to people. *São Paulo* is a political locale where such organizations are located. Hence, a sentence like (1), where the NP *São Paulo* is the subject of the verb *oferecer “offer,”* invites the inference that the social organizations operating in this city are being referenced. *São Paulo* stands out in relation to the social organizations because they are too many, too varied and not necessarily well known, to be useful for the purpose in this context. Thus, we assume that the political locale stands for the social organizations in this sentence.

As presented in section 2, framenet analysis revolves around Lexical Units. A sentence like (1), could be annotated for the following LUs: *oferecer.v “offer,”* evoking the Offering frame; *bar.n “pub,” restaurante.n “restaurant,” comida de rua.n “foodtruck,” cantina.n “cafeteria,”* and *pastelaria.n “dumpling store”* evoking the Food_services frame; and *gosto.n “taste”* evoking the Sensation frame. Note, however, that *São Paulo* would not be considered an LU evoking the Political_locale frame, because framenet annotation does not take proper nouns as targets. Therefore, the only means to extract contextual information about the named entity *São Paulo* in (1) is the linguistic annotation of *oferecer.v*, as shown in (2).

(2) [São Paulo_OFFERER_] oferece^Offering^ [bares, restaurantes, comida de rua, cantinas e pastelarias_THEME_] [para praticamente todos os gostos_POTENTIAL_RECIPIENT_].

**Figure 4:**
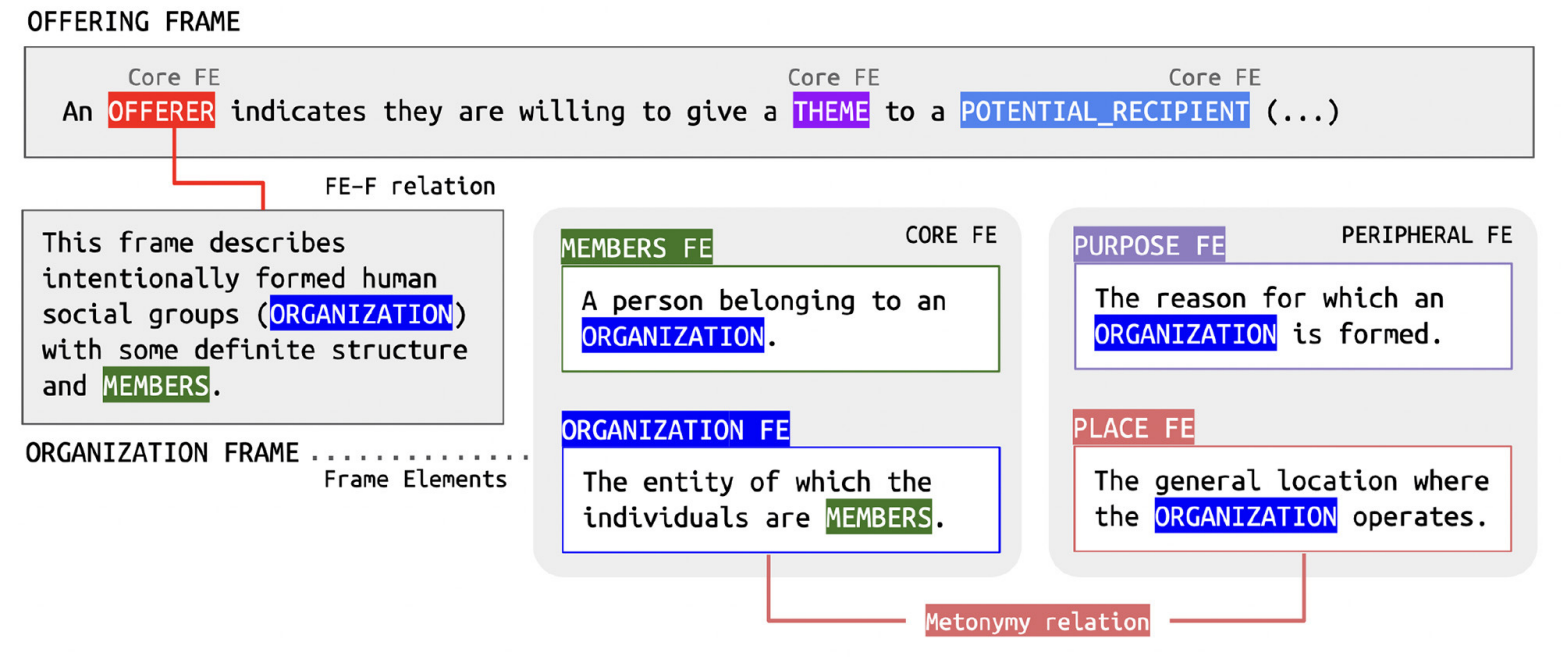
An instance of the Metonymy relation in the FN-Br database.

Metonymic substitutions such as the one exemplified in (2) are very common. To properly represent them in the FN-Br database, a new relation between FEs was proposed. The metonymic mapping of FEs requires two steps. The first is to model, via the FE-to-Frame relation discussed in section 3.1, that the Offerer FE in the Offering frame can be defined by the Organization frame. Through this mapping, we link a frame modeling an event to another one describing an entity. Next, we proceed to the assignment of the metonymic relation in which two FEs of the Organization frame are connected: Organization and Place. Via this relation, we are able to register that the place where an organization is run can be used to reference it, as shown in Figure 4.

The implementation of metonymic relations among FEs covers yet another aspect of contextual information grounded on commonsense knowledge that is not represented in other framenets. Although FE-to-frame and Metonymy relations add other dimensions of representation to the framenet model, they are both restricted to one type of data structure: frames. However, contextual information may also be derived from relations established between LUs. To account for those cases, FN-Br implemented Ternary Qualia Relations, which are presented next.

### 3.3. Ternary Qualia Relations

Deriving a (computational) representation of context from linguistic material—e.g., lexical items in a sentence—involves not only representing the meaning of such linguistic material—whatever definition of meaning is being assumed—but also associating what is linguistically expressed with commonsense knowledge. In general, computational implementations make use of ontologies to represent commonsense knowledge.

In the field of Information Science, ontologies are defined as a formalized explicit specification of the terms in a domain and of the relations between them, given some shared conceptualization (Gruber, 1995; Borst, 1997). In such a definition:

- **formalized** means machine readable;
- **explicit specification** refers to concepts, properties, relations, functions, restrictions, and axioms that are explicitly defined;
- **shared** indicates that the knowledge is grounded on commonsense;
- **conceptualization** refers to some abstract model of a real world phenomenon.

Nonetheless, the use of ontologies for representing the semantics of natural languages is not free of difficulties. Huang et al. (2010) propose an ample discussion of the interface between lexica and ontologies. Meaning associated with words may be, in many cases, characterized as an ontological concept. Also, relations between meanings may be transposed to some extent to relations between concepts in an ontology. However, the kind of knowledge that lexica and ontologies try to capture are different in nature, although the extent of such a difference is not easily measurable.

Such a distinction is even more difficult to delimit in a framenet-like lexicon. In principle, framenets are lexica. However, the motivation for organizing the lexicon in a network of frames is grounded on cognitive and conceptual principles, since the resulting resource is meant to be an implementation of a **semantics of understanding** (Fillmore, 1985). Frames are cognitively inspired and, therefore, relate to the idea of classes in an ontology. LUs, in turn, are disambiguated lemmas, that is, they tend to bear specific meaning, which is a requirement for concepts in an ontology. However, differently from what happens with concepts in an ontology, LUs are also characterized by their use, both in terms of frame evocation and in terms of valency structure, that is, of how FEs are instantiated around them. Crucially, the meaning associated with each LU is grounded on the context of use. Hence, LUs do not have purely intrinsic meanings, such as concepts do. The attempts to ontologize Berkeley FrameNet reveal losses and distortions of the primary goal of such a lexicon (Dolbey et al., 2006; Gangemi, 2010; Scheffczyk et al., 2010).

Nonetheless, if there is an intention to use a framenet for Natural Language Understanding tasks, some degree of formalization is necessary. Fillmore and Baker (2009) state that, to some extent, it is possible to think that each LU evokes its own frame. This is to say that each frame in a framenet, being evoked by several different LUs, actually represents the parcel of meaning that is common to each of them, or in other words, the background knowledge needed for their understanding. Each LU, in turn, bears another parcel of meaning, which distinguishes it from the other LUs evoking the same frame. The solution adopted by FN-Br for creating a lexical ontology is based on an extrapolation of this reasoning.

A lexical ontology seeks to explore the commonalities shared by lexica and ontologies at the same time that it creates mechanisms for reducing the differences between them. According to Lenci (2001), ontologies are formal tools that may represent lexical knowledge, provided that lexical meanings can be treated as entities classified in terms of the types the ontology offers. The relations between meanings would be derived from the relations between the ontological types. Building a lexical ontology involves challenges related to polysemy and context. In other words, the fact that words may bear more than one related meaning and that their meanings depend on the context they are used in poses challenges for formalizing relations between them.

Facing those challenges, the SIMPLE lexical ontology (Lenci et al., 2000), developed within a collaboration funded by the European Union, has the purpose of providing a computational semantic lexicon for twelve languages. Among the ideas implemented in SIMPLE, two of them have been reframed by FN-Br in the implementation of Ternary Qualia Relations: (a) the codification of words as Semantic Units (SemU) and (b) the use of extended qualia relations for characterizing lexical meaning. Simple SemUs are roughly similar to framenet LUs or WordNet synsets. Each SemU expresses a specific—or disambiguated—meaning of the lexical item and is associated to a semantic type specified by the ontology, in a way similar to how LUs are associated with frames. Each semantic type in the ontology has a structure of associated qualia relations.

Extended qualia relations implemented the qualia structure proposed in the Generative Lexicon Theory (GL) (Pustejovsky, 1995). GL proposes that lexical meaning is structured by four generative factors, dubbed qualia roles. Each quale captures how humans understand entities and their relations in the world and aims to provide some minimal explanation for the linguistic behavior of lexical items. The four qualia roles are defined as:

- Formal—describes the basic category for the item and provides the information that distinguishes an entity within a larger set inside its semantic domain.
- Constitutive—expresses a variety of relations concerning the internal constitution of an entity.
- Telic—concerns the typical function or purpose of an entity, i.e., what the entity is for.
- Agentive—concerns the origin of an entity, its creator or its coming into being.

The four qualia roles represent the different dimensions in which the meaning of a lexical item may be characterized. This multi-dimensionality is illustrated for the word *pizza.n* in example (3). These distinct dimensions are triggered by different predicates and are important for characterizing the contextual information associated with the lexical item in different sentences.

(3) The kids wanted pizza (wanted to eat—telic)
  This pizza is too difficult (difficult to make—agentive)
  There was pizza all over the living room (substance—formal)
  This whole grain flour pizza is amazing (ingredient—constitutive)

The SIMPLE specification treats each quale as a relation occupying the top of a hierarchy of more specific relations: the extended qualia relations. For example, the *is_a_part_of* extended quale is a specification of the *constitutive* quale. FN-Br reframed the idea of extended qualia relations implemented in SIMPLE to create Ternary Qualia Relations (TQR).

Each TQR is specified by a background frame. Such a specification is implemented by associating one core FE in the mediating frame to each LU in the TQR. Returning to the constitutive quale connecting flour and pizza in (3), the TQR connecting *flour.n* and *pizza.n* in the FN-Br database is mediated by the Ingredients frame so that the Material and the Product FEs are linked to each of the LUs, respectively.

TQRs are used for both formalizing the FN-Br lexicon and to enrich the network of relations in the database. Two important distinctions between TQRs and extended qualia must be noted:

1. SIMPLE specifies qualia relations at the level of Semantic Types. Each Semantic Type defines the relations a SemU associated to it must implement. FN-Br specifies TQRs directly for each LU. TQRs are LU-to-LU relations.
2. SIMPLE implements binary qualia relations, associating two SemUs. FN-Br implements ternary relations, so that the relation between two LUs is mediated by a frame that represents the meaning of the relation.

**Figure 5:**
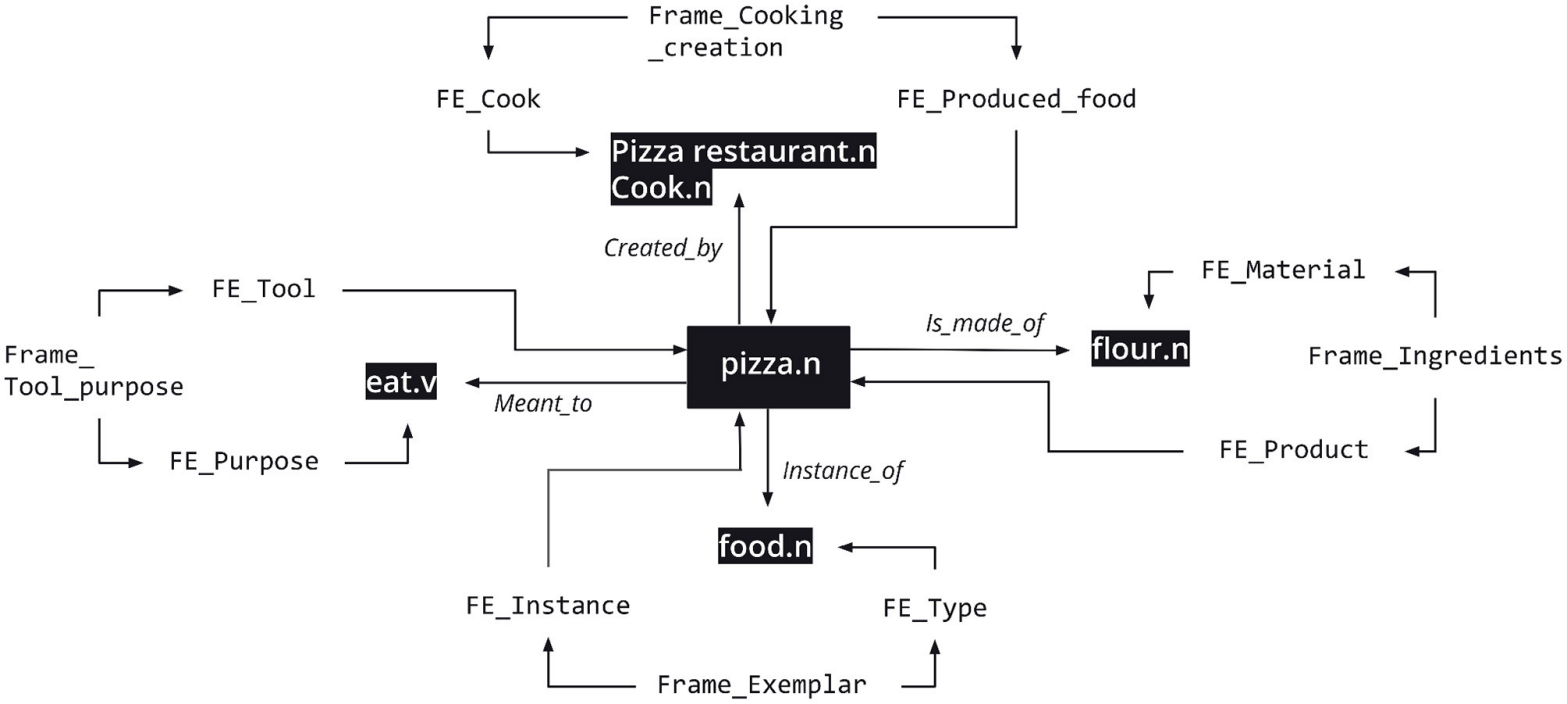
Ternary Qualia Relations for *pizza.n*. Reproduced with permission from Belcavello et al. (2020). (CC-BY-NC).

Instead of proposing an *ad-hoc* label for specifying the quale, TQRs rely on the existing semantic structure of frames to do that. The resulting database structure provides a whole new dimension for analyzing the meaning of a given lexical item in context: for any given sentence, FN-Br is able not only to represent the frames directly **evoked** by the LUs in it, but also the frames **invoked** by the TQRs holding between the LUs, including the specification of which core FEs in the mediating frame are associated with each LU. In other words, there's more information available not only for the disambiguation of polysemous lexical items, but also for representing context and allowing for inferences. By substituting the qualia relations in (3) by TQRs, the specification of the various dimensions of the meaning of *pizza.n* can be represented by means of Figure 5.

The new relations proposed by FN-Br add new dimensions of representation to the model and, when adequately processed—see section 5—may allow for the extraction of contextual information. Before looking into their potential contribution to language processing and understanding tasks, let us first turn our attention to another dimension of context represented in FN-Br: that grounded on other semiotic modes.

## 4. A Multimodal FrameNet: Language Meets Image

In the previous section, we proposed three additional relations to enhance the capacity of FN-Br to represent contextual information. All of them capture two of three dimensions of context proposed by Schifrrin (1994): commonsense knowledge and information in the surrounding text. Situational context is not addressed by any of the new relations, though. Previous research led by framenet teams has shed light on the possibilities and limitations of the model to represent interactional and/or pragmatic frames (Ohara, 2018; Czulo et al., 2020). Both groups propose analyses where grammatical constructions would evoke frames representing situational context. Nonetheless, framenets still lack a consistent set of frames for representing this dimension of contextual information.

**Figure 6:**
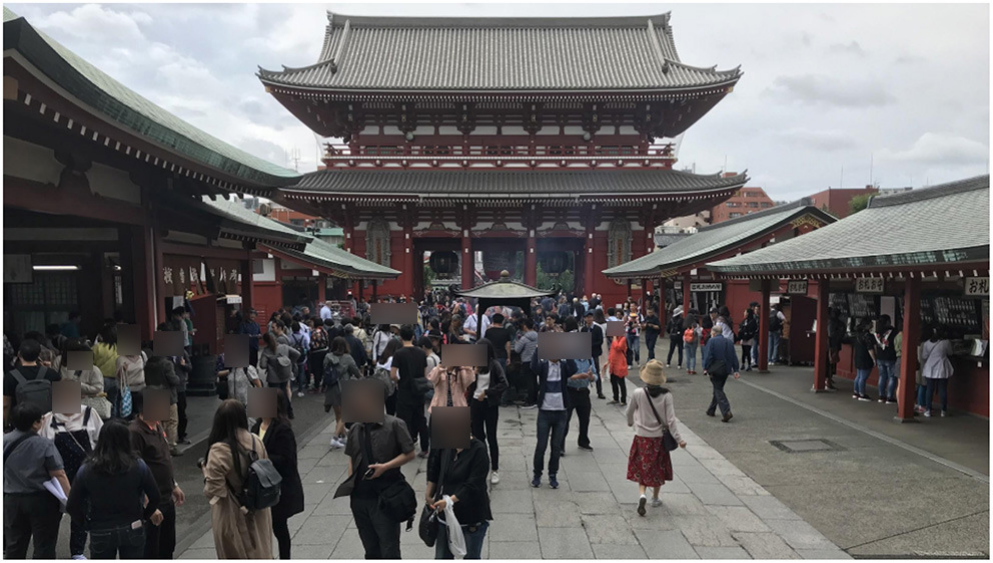
A picture that evokes the Touring frame. Image source: Authors.

FN-Br approaches the question of situational context by using semiotic modes other than verbal language as proxies for assessing how frames expressed in verbal language correlate to those triggered by pictures and videos, for example. According to Belcavello et al. (2020), the hypothesis is that similarly to the way in which words in a sentence evoke frames and organize their elements in the syntactic locality accompanying them, visual elements in photos, video shots, and video sequences may, also, (i) evoke frames and organize their elements on the screen or (ii) work complementarily with the frame evocation patterns of sentences associated with the images—captions, audio, letterings, etc. As an example of (i), consider the picture in Figure 6.

In Figure 6, we see a Place, where there is a building—an exemplar of Japanese architecture—and there are dozens of people, many of them taking pictures. If we consider that these people are Tourists and the building is an Attraction, this scene could be said to evoke the Touring frame. Actually, the idea of a scene by itself is extremely visual, once it is popularly recognized as the fundamental narrative sequence in theater and cinema. Hence, the association between a visual scene and the concept of scene used by Fillmore (1977) in the slogan “meanings are relativized to scenes” is quite straightforward.

The combination of different communicative modalities is a defining characteristic of human expression. Recent work in Computational Linguistics and Computer Vision has resulted in datasets (Lin et al., 2014; Young et al., 2014; Plummer et al., 2015; Elliott et al., 2016; Kuznetsova et al., 2018; Sharma et al., 2018; Changpinyo et al., 2021), models (Li et al., 2019, 2020a; Su et al., 2019; Tan and Bansal, 2019; Chen et al., 2020; Qi et al., 2020; Radford et al., 2021; Wang et al., 2021; Zhang et al., 2021), and metrics (Vedantam et al., 2015; Anderson et al., 2016) focused on multimodal processing. Nonetheless, fine-grained analyses of the semantics yielded by the combination of visual and textual modalities are still rare.

**Figure 7:**
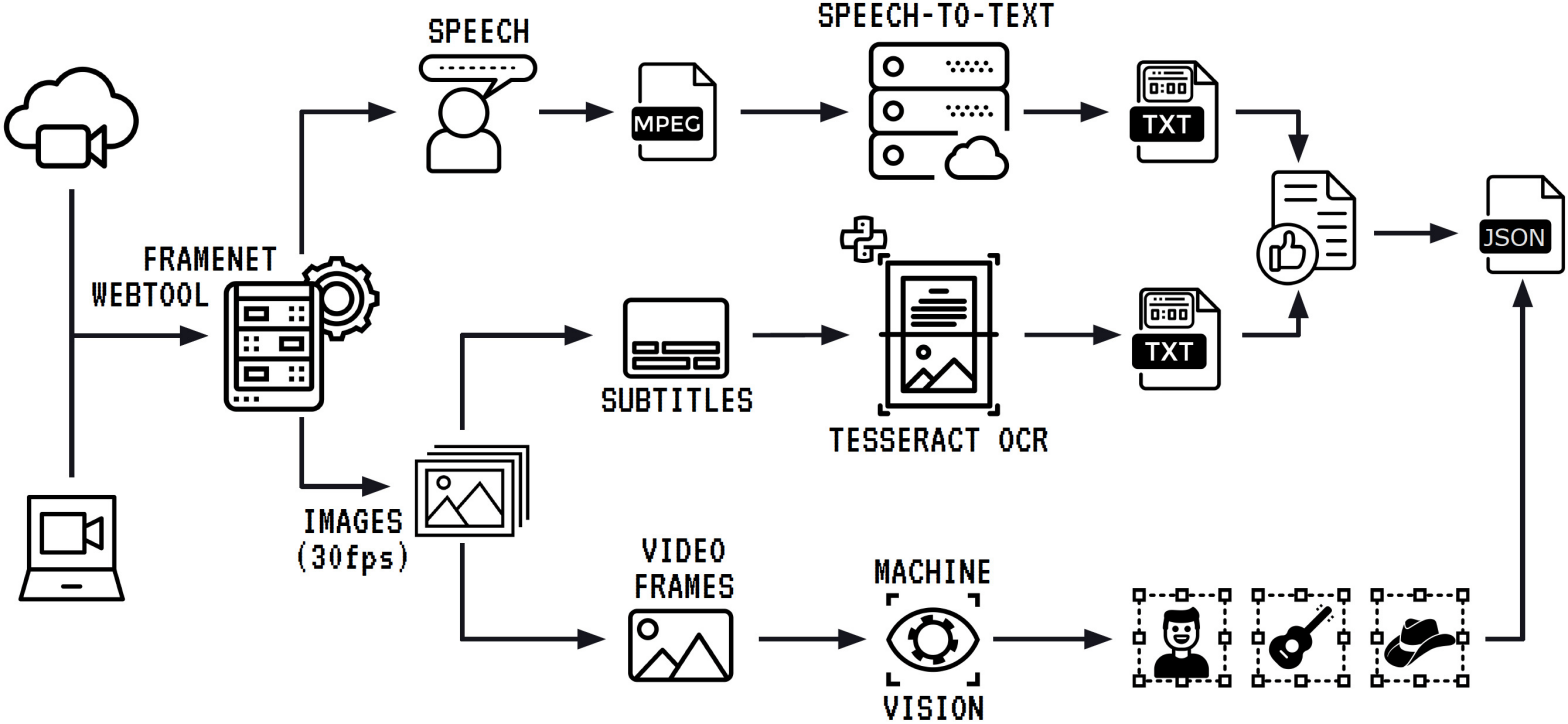
Multimodal corpus import and processing pipeline.

To include the possibility of analyzing other communication modes within the framenet methodology, FN-Br has developed Charon, a multimodal annotation tool and database management application. The tool implements a pipeline for preprocessing multimodal corpora that includes the steps in Figure 7.

The process begins with the video input, either from a web address or a local file. The tool separates audio data and image data to proceed with parallel tasks. The extracted audio runs through a speech-to-text cloud service, which transcribes all the spoken audio into text. The transcription is organized by sentences, and each of them receives a correspondent time stamp. To finish the processing of textual data, the transcription needs to be combined with the content extracted from subtitles that may appear in videos where more than one language is used. For instance, in the pilot multimodal annotation experiment reported in Belcavello et al. (2020), the corpus used was an episode of a Brazilian Travel Show where the host speaks Brazilian Portuguese when talking to the camera, and English when interviewing locals. The interviews are subtitled in Brazilian Portuguese. Although the corpus used in the pilot annotation experiment refers to the Tourism domain, any frame can be used for multimodal annotation, in principle.

The process of text extraction from subtitles starts in the image processing segment of the pipeline. First, the video is converted into still images, at a 30 frames per second rate. Then, these images are submitted to an OCR service which extracts the subtitles and timestamps them. The text generated is combined with the audio transcription and this combined text file is offered to a human annotator who can review and edit it. The final version of the text is sent to the output file. The third segment of the pipeline takes the still images, runs them through a computer vision algorithm, which tags relevant objects in each frame, timestamps them and sends all this information to the output file.

At the end of the pipeline, we have a JSON file that contains: (i) the text generated from the combination of audio and subtitles with start and end timestamps for each sentence; and (ii) coordinates of all detected visual objects and the start and end timestamps of their appearances on screen.

**Figure 8:**
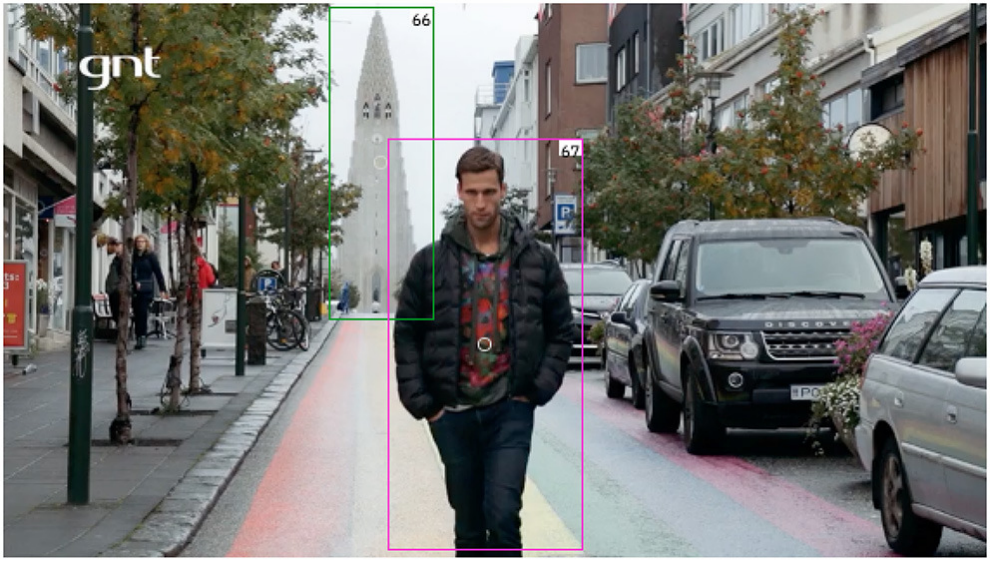
Example of video annotation in Charon. Image reproduced with permission from GNT.

After, the data is made available for the following annotation methods: (i) independent text annotation; (ii) independent visual annotation; (iii) text-oriented multimodal annotation; and (iv) visual-oriented multimodal annotation. In Figure 8, we see an example of text-oriented visual annotation. Both objects—66 and 67—were annotated for the Attraction_tourism frame. Object 66, the Reykjav Cathedral, instantiates the Attraction FE, while object 67, the TV host, instantiates the Tourist. This annotation is text-oriented because seconds before this video frame, while other image sequences are shown, the host says the sentence in (4).

(4) O centro de Reykjavik é famoso pela arte de rua, pelas casas coloridas e por algumas atrações turísticas.
  *Downtown Reykjavik is famous for street art, colored sidewalks and for some tourist attractions*.

This sentence made it possible for the text annotator to choose the Attraction_tourism frame for the multiword expression *atrações tur*í*sticas.n* (*tourist attractions*). Consider, then, the situational context. When a viewer sees the shot depicted in Figure 8 it is natural to recognize the cathedral shown in the previous shot. This second appearance of the cathedral, now in the background, instantiates the Attraction FE. At the same time, in the foreground we have the host figure, which instantiates the Tourist FE. With these two FEs annotated, we can say we have the Attraction_tourism frame in the image matching the Attraction_tourism frame evoked by the multiword expression *atrações tur*í*sticas.n* (*tourist attractions*).

This is one example of how Charon allows for the enrichment of the FN-Br database with multimodal data. The possibilities allowed for the inclusion of other communicative modes in framenet made plans for building fine-grained semantically annotated multimodal datasets possible. Such plans are presented next.

### 4.1. The Frame^2^ Dataset

This dataset aims to provide a means to analyze how the frame-based semantic representation of verbal language interacts with that produced by the frame-based annotation of video sequences, or, more precisely, of sequences of visual frames forming a video. The effort is aimed at detecting audio and video combination possibilities in terms of frames, as in the example shown in Figure 8.

Because the annotation of video sequences in correlation with linguistic data adds a whole new dimension of meaning construction possibilities, the Frame^2^ dataset was planned as a domain-specific dataset for the Tourism domain. The modeling of this domain in the FN-Br database counts with all the additional dimensions described in section 3. Hence, the multimodal objects selected for annotation are the episodes of the TV Travel Series “Pedro pelo Mundo.” The show premiered in 2016 on GNT, a cable TV channel dedicated to entertainment and lifestyle productions. Four seasons of “Pedro pelo Mundo” were aired until 2019. There were 40 episodes in total. The first season has 10 episodes of 23 min each. The second, third and fourth are also composed by 10 episodes each, but these are 48 min long. For the first data release of Frame^2^, we plan for the annotation of all episodes in the first season.

The plot of each episode focuses on getting in contact and exploring social, economic and cultural aspects of a location which has experienced some kind of recent transformation. Thus, what the viewer sees is Pedro Andrade, the host, trying to connect with locals, instead of merely proposing a touristic view of popular places of interest. Therefore, most of the episodes focus on a specific city, like Edinburgh, but some propose a broad view of a country, like Iran, Colombia and the already mentioned example of Iceland.

The format of the show combines stand ups, voice-over sequences, short interviews and video clip sequences. It offers, then, rich material as exemplar of complex composition of audio and video for meaning making. For each 23-min-episode, the audio transcription combined with the subtitles according to the pipeline in Figure 7 generates approximately 200 sentences, which means 2,000 sentences for the first season. Taking the FN-Br average of 6.1 annotation sets per sentence, the annotation of the whole verbal language part of the corpus should yield, when complete, about 12,200 annotation sets. For the video annotation, the pilot experiment yielded an average of 2.5 annotation sets per sentence, meaning an expected total of circa 5,000 annotation sets upon completion of the annotation process.

All ten episodes were submitted to Charon's import and processing pipeline. The resulting text from both speech-to-text and OCR have been then reviewed, edited and compiled by trained annotators. Once this stage is finished, the sentences are ready for annotation.

**Figure 9:**
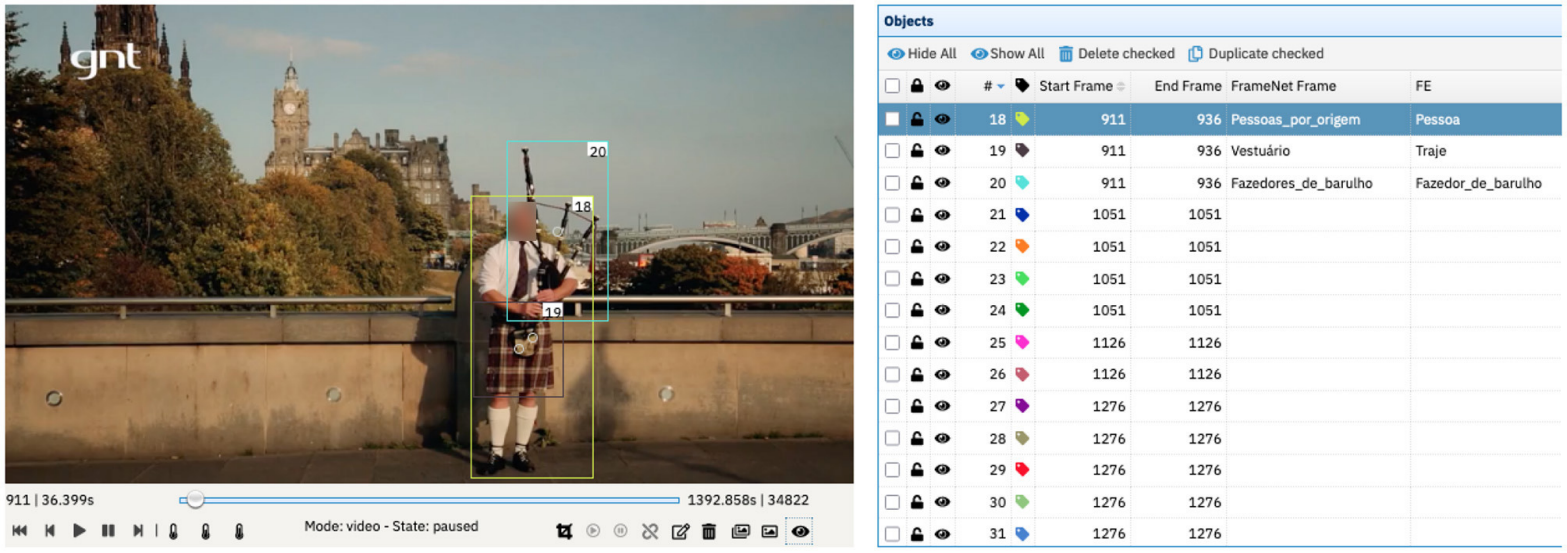
Example of video annotation for frames and FEs in Charon. Streetscene image reproduced with permission from GNT.

In the next step, one annotator manually annotates the sentences of one episode, using the FN-Br Web Annotation Tool and following FrameNet's guidelines for full-text annotation (Ruppenhofer et al., 2016). After this, the same annotator starts the annotation of the visual elements present in the same episode of the corpus, oriented by the already concluded text annotation. As previously mentioned, adopting a text-oriented annotation means that the annotator looks for the evocation of the same or correlate frames annotated in the text, now in the image–as shown in Figure 9.

Nonetheless, the annotation of both modes—audio and video—does not always generate direct correlations. In episode 6 of the corpus, when visiting Edinburgh, the host says the sentence in (5).

(5) Quando a gente pensa na Escócia, a primeira coisa que vem à mente é homem de saia, uísque escocês e gaita de fole.
  *When we think of Scotland, the first things that come to mind are man in skirts, Scotch whisky and bagpipe*.

For this sentence the annotator chose the frame People for *homem.n* (*man.n*). It is spoken some seconds before the viewer sees the image in Figure 9.

As Figure 9 shows, the annotator chose People_by_origin as the frame evoked by object 18, instead of the People frame evoked by *homem.n* (*man.n*). The reason behind this choice is the fact that the man depicted in the video right after the audio mentions *homem de saia* (*man in skirt*) is wearing a kilt and playing a bagpipe. These represent the typical clothing and musical instrument of Scotland, respectively. This combination of factors makes it very likely to infer that what the viewer sees is a Scottish person, a Scot. Therefore, it makes it possible for the annotator to choose the People_by_origin frame instead of the People frame. This kind of annotation is an example of a multimodal frame-mediated Ternary Qualia Relation.

**Figure 10:**
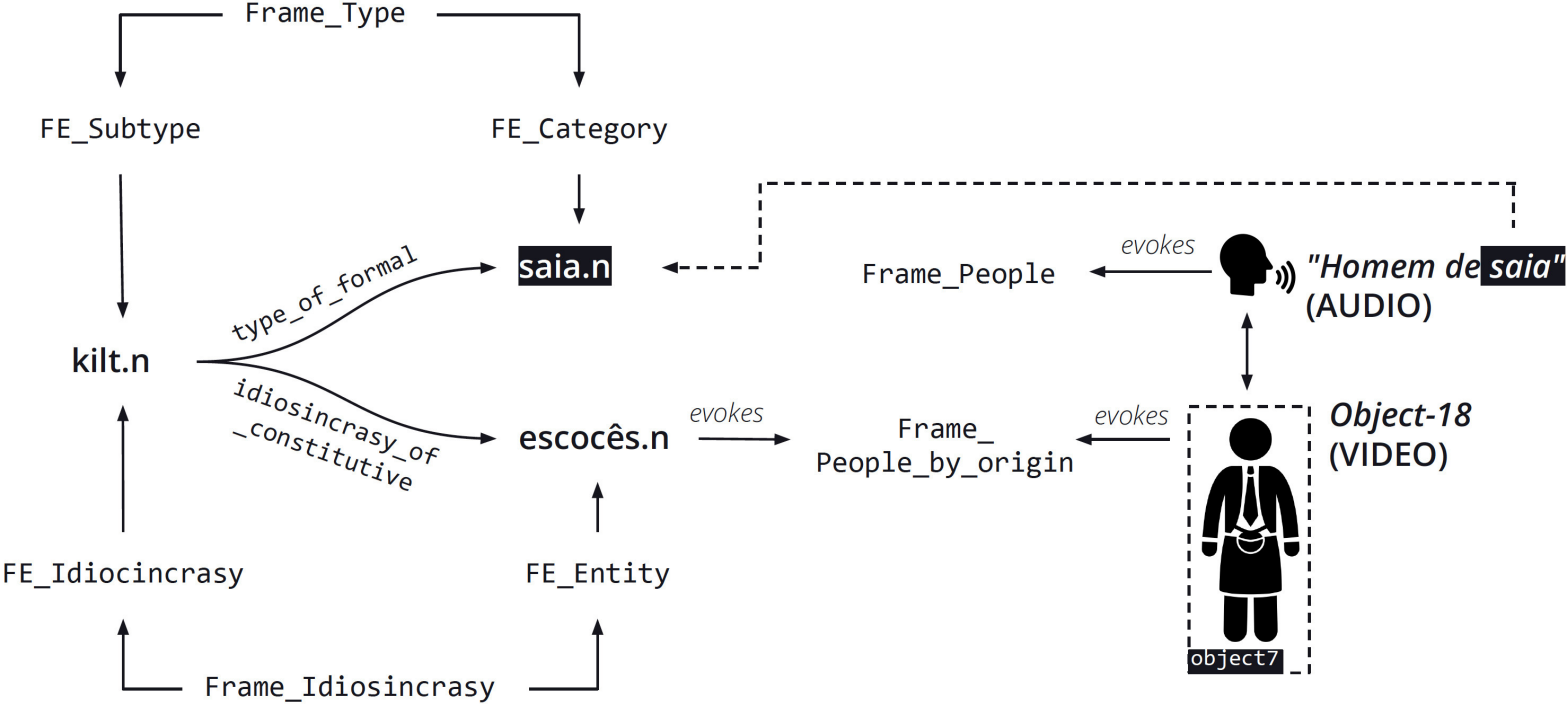
Frame-mediated ternary qualia relations for homem de saia.n and object 18. Reproduced with permission from Belcavello et al. (2020). (CC-BY-NC).

Figure 10 shows a schematic representation of the relations in action. First, a subtype of the formal quale, mediated by the Type frame connects the LUs *kilt.n* and *saia.n* (*skirt*) in the FN-Br database. Second, a subtype of the constitutive quale mediated by the Idiosyncrasy frame connects the LU *kilt.n*, instantiating the Idiosyncrasy FE to the LU *escocês.n* (Scot), instantiating the Entity FE in this frame. Finally, the LU *escocês.n* evokes the People_by_origin frame, which is precisely the one evoked by object 18 in Figure 9.

### 4.2. The Framed Multi 30k Dataset

Despite the amount of recent work focusing on the development of multilingual image description datasets (Elliott et al., 2017) and the significant advances obtained by research on Multimodal Machine Translation and Crosslingual Image Description (Specia et al., 2016; Elliott, 2018; Lala and Specia, 2018; Yao and Wan, 2020), we argue that the further development of computational applications aimed at improving the performance of Machine Translation algorithms requires a multimodal-multilingual dataset that incorporates the type of fine grained semantics only made possible by grounding textual references to specific image regions via the establishment of textual-visual frame relations. In order to develop this frame-based multimodal application, our proposed model must be able to not only attribute categories to objects in an image, but also to take advantage of the relations that are established between those entities, reflecting how they interact with the world, taking into account aspects like the background scenario evoked by that particular visual scene.

Current work in the field of Multimodal Machine Translation has been focusing on expansions of the popular dataset for sentence-based image description Flickr30k (Young et al., 2014). This dataset consists of 31,783 images of everyday activities, events and scenes, each of them independently captioned by five annotators who were not familiar with the specific entities and circumstances depicted, resulting in conceptual descriptions (Hodosh et al., 2013) that focus exclusively on the information that can be obtained from the image alone. The Multi30k dataset (Elliott et al., 2016)—a multilingual extension of Flickr30k—extends the Flickr30K dataset with German translations created by professional translators over a subset of the English descriptions, and new German captions crowdsourced independently of the original English captions. The Flickr30k Entities (Plummer et al., 2015) augments the original captions from Flickr30k with 244,000 coreference chains—linking mentions to the same entities in images—and 276,000 manually annotated bounding boxes corresponding to each entity.

To combine the data from both Flickr30k Entities and Multi30k datasets with the network of frames and qualia relations described in the previous sections, we start by creating five new Brazilian Portuguese descriptions and five English-Portuguese translations for each of the 31,014 images and correspondent English captions in the Flickr30k dataset. For the Brazilian Portuguese caption creation task, undergrad students from the Federal University of Juiz de Fora who are native speakers of Brazilian Portuguese are presented with the image and write a caption for it following the guidelines in Hodosh et al. (2013). For the translation task, annotators are presented with an image alongside one of its original English descriptions, making it possible for students majoring in translation studies to provide the correspondent translated description.

**Figure 11:**
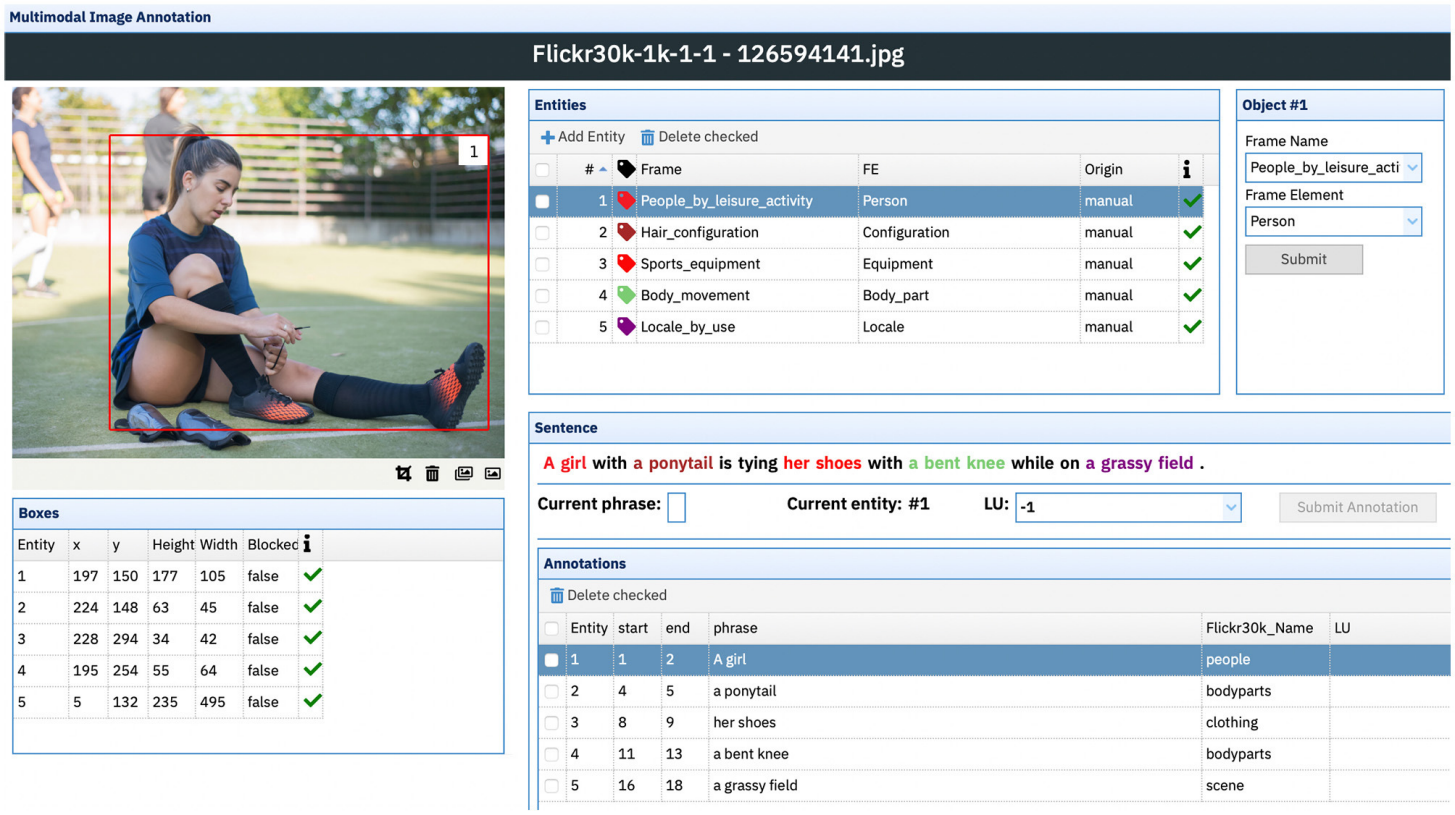
Multimodal image annotation interface. Due to copyright issues, the original image from the Flickr 30K corpus has been substituted by a similar licensed image from Adobe Stock. Image reproduced from Adobe Stock with permission.

The task of enriching the multimodal dataset with fine-grained semantic information, provided by the extensive network of relations from FN-Br, is achieved by using Charon. First, all captions are automatically pre-processed by DAISY (as described in section 5.1) to identify frame-evoking LUs that may match object classes from the images. If a given entity from the image has, for example, the class “people” assigned to it, DAISY automatically correlates the bounding box corresponding to that object with the frame People in the FN-Br database. Once this process is completed, all images and their respective captions are loaded into the annotation tool, where annotators can use the Image Annotation interface (Figure 11) to visualize: (i) each image, with its respective entities and bounding box information extracted from the Flickr30k Entities dataset; (ii) one of its five captions, with highlighted sentence fragments linked to a manually annotated bounding box; and (iii) the frames and FEs evoked by each textual-visual pairing of a sentence fragment and its correlated object from the image.

Example sentence (6) has five highlighted segments—“A girl,” “a ponytail,” “her shoes,” “a bent knee,” “a grassy field”—each one corresponding to five entities/bounding boxes in the image—numbered from 1 to 5 in the Entities, Boxes and Annotations windows. For the segment “A girl,” correlated with the Entity 1, DAISY—while considering the class “people” from the original dataset—automatically assigned the frame People. Nonetheless, when taking into account both the visual and textual modalities simultaneously, the annotator can decide that, in this scenario, the correct frame is People_by_leisure_activity. The same goes for the segment “her shoes” which, without the information provided by visual modality, could be considered an LU from the Clothing frame, but was correctly tagged by the annotator for the Sports_equipment frame given the fact that, as the image shows, the shoes are soccer cleats.

(6) A girl in a ponytail is tying her shoes with a bent knee while on a grassy field.

After the completion of the annotation process, the resulting frame-based multimodal dataset is expected to have 155,070 original Brazilian Portuguese captions, 155,070 new English-Portuguese translated captions, and five sets of Entity-FE-frame relations for each one of the 276,000 manually annotated bounding boxes corresponding to each entity.

## 5. Experiments

In this section, we present two experiments to evaluate the extent to which the representation of both sentence cotext and commonsense knowledge provided by the FE-to-frame and the Ternary Qualia Relations may aid in Computational Linguistics tasks. Section 5.1 reports on the task of automatically identifying frame-evoking LUs in sentences, while section 5.2 discusses the application of the multidimensional FN-Br representation to domain adaptation in Neural Machine Translation (NMT).

### 5.1. Automatic Identification of Frame-Evoking LUs

Automatically identifying frame-evoking LUs is typically the first step in any automatic Semantic Role Labeling (SRL) process built on a framenet. Semafor (Chen et al., 2010), Open Sesame (Swayamdipta et al., 2017) and Sling (Ringgaard et al., 2017) are the most known examples of frame-based semantic role labelers. Although using different computational techniques, the three of them rely on the BFN annotated corpus for training. The data driven approach adopted by those systems makes it virtually impossible for them to be expanded to other languages, because no other framenet for no other language has annotated as many sentences as BFN: 200,000.

Given this limitation, FN-Br developed DAISY (Disambiguation Algorithm for Inferring the Semantics of Y). DAISY differs from the systems mentioned in the previous paragraph in two ways: (i) the assignment of a frame to an LU relies on the structure of the network of frames, FEs and LUs in the databse, without using information from annotation sets and (ii) because it is annotation-independent, it can be used for any language for which there is a framenet, regardless of the number of annotation sets available.

DAISY's algorithm builds a graph from the several relations available in FN-Br. Such a graph can be regarded as a type of semantic network whose nodes are word forms, lexemes, LUs and frames. The construction of the graph includes the following steps:

1. The syntactic structure of the sentence is obtained via a dependency parser. For the experiment reported in this article, we used Universal Dependencies (UD) tags (de Marneffe et al., 2021) automatically obtained from the Spacy UD library^2^. This process also provides the lemmas to be used in the graph.
2. The system searches the sentence for multiword expressions in the FN-Br database of lemmas.
3. Based on the dependency tree, the system builds clusters of lemmas. Each cluster contains a lemma and the lemmas that establish a dependency relation with it.
4. LUs associated with each lemma are obtained from the FN-Br database.
5. The frame evoked by each LU is obtained from the FN-Br database;
6. The frame-to-frame relations are retrieved and stored.
7. The FE-to-Frame relations are retrieved and stored as relations between frames;
8. All relations above are implemented as arches in the graph.

**Figure 12:**
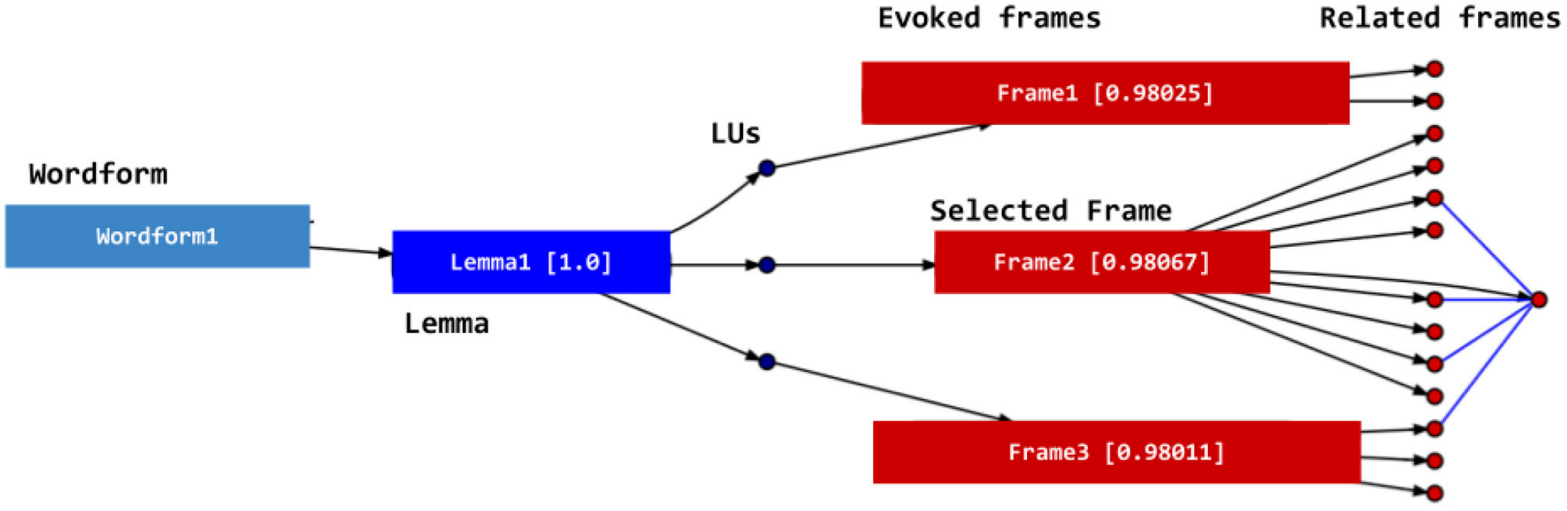
The spread activation process in DAISY.

Using the graph, DAISY can not only assign frames to lexical items when a given lemma evokes only one frame, but also choose among several options the best frame for the lemma, given the sentence context. The disambiguation is performed through the attribution of a value to each node. The system uses spread activation (Diederich, 1990; Tsatsaronis et al., 2007) in this process: each candidate lemma receives an initial value (e.g., 1.0). Those values spread from those nodes and decay each time they move to another node. The sum of the values arriving at each node is calculated using a logistic function that calculates the value that is to be spread to the neighboring nodes. This spreading process reaches the nodes representing the frames and backpropagates to the LUs evoking those frames. At this point, the final value for each LU is calculated. LUs with the highest values are chosen for each lemma, together with the frame they evoke. This process is summarized in Figure 12.

In this article, we use DAISY to evaluate the contribution of FE-to-frame relations for representing sentence level context. To do that, we randomly picked 5,000 Brazilian Portuguese sentences from the FN-Br database that were annotated by trained lexicographers, and 5,000 English sentences also annotated in the same way. We submitted the unannotated version of those sentences to DAISY and had them automatically tagged for frame-evoking LUs in both languages. Next, we compared the results between the two languages. Experimental results are shown in Table 1.

**Table 1:** Evaluation of Daisy, using the FE-F relation in Brazilian Portuguese and English for the assignment of frames to lemmas.

**Language**	**Correctly assigned**	**Wrongly assigned**	**Non-assigned**
Brazilian Portuguese	59.1%	30.1%	10.8%
English	55.8%	27.1%	17.1%

Results in Table 1 allows for some relevant conclusions. First, the very similar performance of the algorithm in both Brazilian Portuguese and English demonstrates that DAISY can be used for automatically assigning frames to lemmas in any language for which there is a framenet, regardless of the number of annotation sets in that language. This is especially relevant in a context where the Multilingual FrameNet data release is made available^3^. This represents a tremendous gain for frame-based automatic semantic role labeling for languages other than English, since all the other currently available tools require training on a large amount of annotated data.

Second, the percentage of non-assigned frames reveals there is room for improvement of DAISY's performance. Non-assignment is mostly due to database errors—such as a lacking word form, for example—and mismatches between the part of speech of the lemma recognized by the dependency parser and the one in the framenet database.

Finally, because DAISY is not implemented as a black box machine learning system, the cases where frames have been wrongly assigned can be analyzed in great detail, leading to potential improvements in the system architecture. The inclusion of TQRs, for example, may lead to sensible improvement in the results, since many cases of incorrect frame assignment are due to very minimal score differences between candidate frames. Currently, the FN-Br database does not have TQRs implemented for all LUs in all frames, but they do exist for the frames in the Sports domain (Costa, 2020). Next, we evaluate the role of TQRs in another task relying on sentence-level context: that of domain adaptation in machine translation.

### 5.2. Domain Adaptation in NMT

Suitability to context is one of the key aspects of quality estimation for machine translation. Assessing the output of an MT system for adequacy—that is, for the preservation of the meaning of the input sentence—necessarily involves analyzing context. Domain adaptation is the Natural Language Processing task aimed at improving the adequacy of a machine translated sentence for a specific context. Chu and Wang (2018) survey the methods commonly used for this task. Most of them use fine tuning to adapt the performance of the systems to a specific domain, by providing neural nets with in-domain data. However, as pointed out by Khayrallah et al. (2018) and Thompson et al. (2019), once NMT models are fine-tuned for a specific domain, their performance on out-of-domain translations decays sensibly.

To propose a solution for domain adaptation in NMT that does not require in-domain training data and fine tuning, at the same time that it does not compromise the NMT system's performance for out-of-domain translation, FN-Br developed Scylla, a post editing pipeline for domain adaptation using frames and qualia (Costa et al., 2022). Scylla relies on a version of DAISY that implements TQRs—on top of frame-to-frame and FE-to-frame relations—to automatically assign frames for the lexical items in both the source sentence submitted to a commercial NMT API and the target sentence automatically translated by such an NMT API^4^.

The reason why we chose the experiment reported in Costa et al. (2022) to discuss the contributions of TQRs for representing contextual information in FN-Br is because, unlike FE-to-frame relations, which have been already implemented for all the frames in the database, TQRs have only been fully implemented for the circa 40 frames modeling the Sports domains in FN-Br (Costa, 2020). Also, the frames proposed by Costa (2020) are bilingual, meaning that there are LUs evoking them for both Brazilian Portuguese and English.

Scylla compares the frames evoked in both the source sentence and the automatically translated sentence and, if they do not match, the system substitutes the inadequate term by another one that is adequate given the sentence-level context. According to Costa et al. (2022), the process can be described as follows:

1. The source sentence is sent to the NMT API and the n-best generated translations are retrieved.
2. The system queries the bilingual FN-Br database of Sports frames and a bilingual dictionary to retrieve all possible translations for the lexical items in the n-best translations^5^.
3. The system compares the translation equivalents retrieved with the lexical items in the source sentence and creates an alignment pair for every match in the comparison.
4. The equivalence sets are concatenated to generate a set of translation alternatives for each lexical item in the sentence.
5. Whenever the equivalence set does not match the translation generated by the NMT API, Scylla substitutes the out-of-context translation by an in-domain equivalent.

**Figure 13:**
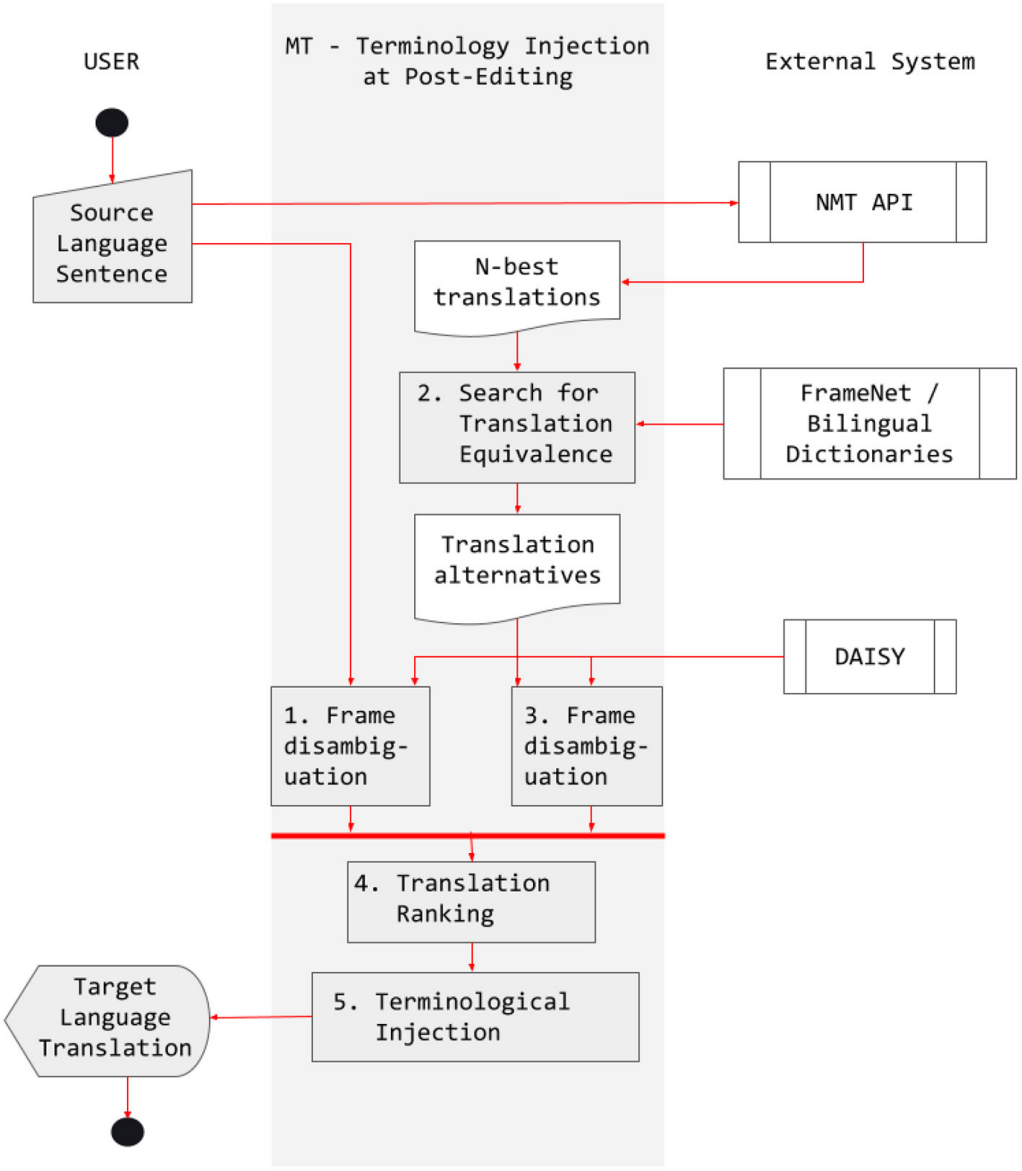
The Scylla Pipeline. Reproduced with permission from Costa et al. (2022). (CC BY-NC-ND 4.0).

The Scylla pipeline is summarized in Figure 13. To illustrate how the system works with a real example, consider sentences in (7)–(10).

(7) O ponta é o jogador que menos tempo tem para pensar na armção de uma jogada.
*Source sentence*

(8) The winger is the player with less time to think about setting up a strike.
*Gold standard translation*

(9) The **forward** is the player who has less time to think about setting up a move.
*Baseline system translation*

(10) The **winger** is the player who has less time to think about setting up a play.
*Scylla translation*

The sentence in (7) was translated in (8) by a professional human translator. The baseline system generated the sentence in (9) for the same source sentence. Note that there are two lexical differences between (8) and (9): *winger.n* vs. *forward.n* and *strike.n* vs. *move.n*. While the difference in the second pair does not substantially alter the meaning of the sentence, the one in the first pair does, because forward and winger are different positions in soccer. While the first is usually positioned closer to the opponent's goal area, the second makes the link between mid-field and the attacking zone. Because of the TQR relations modeled in the FN-Br database for the Sports domain, such a difference derived from commonsense knowledge can be taken into consideration by Scylla for performing a lexical substitution in the sentence translated by the NMT API, changing *forward.n* to *winger.n*.

To evaluate the performance of Scylla, Costa et al. (2022) conducted a domain-specific sentence translation experiment for the Brazilian Portuguese–English language pair. Scylla was evaluated against the commercial NMT API, which was taken as the baseline, for three metrics: BLEU (Papineni et al., 2002), TER, and HTER (Snover et al., 2006).

For the experiment, Costa et al. (2022) put together a domain-specific dataset comprising:

- 50 sentences in Brazilian Portuguese for the Sports domain, extracted from authentic texts. Each sentence had at least one polysemous lemma with at least two possible meanings, one of which related to the Sports domain.
- 50 reference translations into English of the source Brazilian Portuguese sentences, translated by professional translators who were native speakers of English and familiarized with the target domain.

The 50 Brazilian Portuguese sentences were submitted to both the baseline system and to Scylla and the automatically translations into English were evaluated for BLEU, TER, and HTER. Results reported by Costa et al. (2022) are reproduced in Table 2.

**Table 2:** Evaluation of the baseline and Scylla systems for BLUE, TER, and HTER.

	**Baseline**	**Scylla**
BLEU (higher is better)	53.13	53.66
TER (lower is better)	36.23	36.47
HTER (lower is better)	13.80	**7.38**

Note that the performance of both systems is very similar for both BLEU and TER, which are automatically calculated. However, Scylla's performance for HTER—boldfaced in Table 2—surpasses that of the baseline system by almost 47%. BLEU (Papineni et al., 2002) measures correspondences between n-grams in the translated sentences and in the gold standard translations. For this metric, the higher the score, the better the translation. TER (Snover et al., 2006) computes the minimal editing effort needed to make machine translated sentences match gold standard translations. Hence, for this metric, the lower the score, the better the performance. Both BLEU and TER are form-based metrics, meaning they capture neither the fact that a small change in one or two n-grams can substantially affect the quality of a translation, or the fact that different lexical choices may be equally adequate in a translation context, respectively.

HTER (Snover et al., 2006), however, is calculated based on edits made to translated sentences by professional human translators, who are instructed to make as few changes as possible so that the automatically translated sentence becomes fluent and equivalent in meaning and context to the reference translation. The HTER methodology recommends having three professional translators make the edits and calculate the editing effort based on the average of edits per sentence, reducing subjectivity.

The fact that HTER relies on professional human translators ensures that the analysis of each sentence translated by either the baseline or Scylla systems is evaluated for its domain—or contextual—adequacy. According to the analysis by Costa et al. (2022), professional translators keep track of the different equivalence possibilities that can be found between languages. Moreover, since the sentences in the source-language corpus were collected from authentic texts, translators could easily identify them as belonging to the Sports domain. Therefore, the fact that Scylla outperforms the baseline system with a 47% improvement in HTER strongly supports the claim made in this article that the FN-Br multidimensional semantic model, which is enriched with FE-to-frame and Ternary Qualia Relations, provides a useful representation for contextual information at the sentence level.

## 6. Conclusions

In this article, we discussed the extent to which the framenet model is capable of representing contextual information of three types: sentence cotext, commonsense knowledge and situational framing. We started by presenting the limitations of the original BFN model for representing any dimension of context different from the fragments of commonsense knowledge captured by frames, their FEs, and the relations between frames. We also pointed out that such knowledge is mostly captured in BFN in an unstructured fashion, and that it is mostly limited to the process of frame evocation.

Next, we presented and discussed three new types of database structure implemented in FN-Br to both enrich the representation of commonsense knowledge and provide a structured means for representing sentence cotext in the database: FE-to-frame, Metonymy and Ternary Qualia Relations. Those new dimensions of meaning and context representation allow FN-Br to capture and represent information derived not only from frame evocation, but also from frame invocation processes. We also discussed how the expansion of the framenet model to the treatment of multimodal data can be used as a proxy for studying extra-linguistic contextual information.

To discuss the potential of such an enrichment of the model, we presented the plans for building two multimodal datasets and reported on two experiments relying on the multidimensional FN-Br database structure for tackling the problems of semantic role labeling and domain adaptation for languages other than English.

Although the new additions to the framenet model presented in this article do not fully address the issues related to frame invocation and situational framing, they represent a sensible advance in the framenet model for the representation of context. In future work, we plan to add new layers of contextual representation to the framenet model, including means for addressing those issues.

## Data Availability Statement

The datasets presented in this study, with an exception made for the multimodal datasets still under construction, can be found in online repositories. The names of the repository/repositories and accession number(s) can be found at: https://github.com/FrameNetBrasil/webtool or in the papers cited as references for the respective datasets.

## Author Contributions

EM, FB, MV, and MG each contributed a section. FB developed the pilot study for multimodal annotation of video sequences. MV developed the pilot study for multimodal annotation of the image-caption pairs. EM and TT are responsible for the automatic frame assignment experiment. AC, MM, EM, and TT are responsible for the domain adaptation experiment. All authors contributed to the article and approved the submitted version.

## Funding

Research presented in this paper was funded by CAPES PROBRAL grant 88887.144043/2017-00. AC and MV research was funded by CAPES PROBRAL Ph.D., exchange grants 88887.185051/2018-00 and 88887.628830/2021-00, respectively. FB research was funded by CAPES PDSE Ph.D., exchange grant 88881.362052/2019-01. MG research was funded by CAPES PROBRAL post-doc exchange grant 88887.387875/2019-00.

## Conflict of Interest

The authors declare that the research was conducted in the absence of any commercial or financial relationships that could be construed as a potential conflict of interest.

## Publisher's Note

All claims expressed in this article are solely those of the authors and do not necessarily represent those of their affiliated organizations, or those of the publisher, the editors and the reviewers. Any product that may be evaluated in this article, or claim that may be made by its manufacturer, is not guaranteed or endorsed by the publisher.

## Footnotes

^1^Torrent, T. T., Matos, E. E., Costa, A. D., Gamonal, M. A., Peron-Corra, S., and Paiva, V. M. R. L. (submitted). A Flexible Tool for a Qualia-Enriched FrameNet: the FrameNet Brasil WebTool. *Language Resources and Evaluation*.

^2^ https://spacy.io/

^3^ https://github.com/icsi-berkeley/framenet-multilingual-alignment

^4^For the experiment reported in Costa et al. (2022), the Google Translate V2 API was used.

^5^For the implementation reported in Costa et al. (2022), the Oxford Dictionary API was used under a free academic license.

## References

[B1] Anderson P. Fernando B. Johnson M. Gould S. (2016). Spice: semantic propositional image caption evaluation. In European Conference on Computer Vision (Amsterdam: Springer), 382–398.

[B2] Baker C. F. Fillmore C. J. Lowe J. B. (1998). The Berkeley FrameNet project. In Proc. of COLING-ACL (Montreal, QC), 86–90.

[B3] Barcelona A. (2002). Clarifying and applying the notions of metaphor and metonymy within cognitive linguistics, in Metaphor and Metonymy in Comparison and Contrast, eds R. Dirven, and R. Prings (Berlin: Mouton De Gruyter), 207–277.

[B4] Belcavello F. Viridiano M. Diniz da Costa A. Matos E. E. D. S. Torrent T. T. (2020). Frame-based annotation of multimodal corpora: Tracking (a)synchronies in meaning construction, in Proceedings of the International FrameNet Workshop 2020: Towards a Global, Multilingual FrameNet (Marseille: European Language Resources Association), 23–30.

[B5] Bender E. M. Gebru T. McMillan-Major A. Shmitchell S. (2021). On the dangers of stochastic parrots: Can language models be too big? in Proceedings of the 2021 ACM Conference on Fairness, Accountability, and Transparency (Toronto, ON), 610–623.

[B6] Bommasani R. Hudson D. A. Adeli E. Altman R. Arora S. von Arx S.. (2021). On the opportunities and risks of foundation models. arXiv [Preprint]. arXiv:2108.07258.

[B7] Borst W. (1997). Construction of Engineering Ontologies (Ph.D. thesis), University of Twente, Enschede.

[B8] Changpinyo S. Sharma P. Ding N. Soricut R. (2021). Conceptual 12m: pushing web-scale image-text pre-training to recognize long-tail visual concepts, in Proceedings of the IEEE/CVF Conference on Computer Vision and Pattern Recognition (Nashville), 3558–3568. 10.1109/CVPR46437.2021.00356

[B9] Chen D. Schneider N. Das D. Smith N. A. (2010). SEMAFOR: frame argument resolution with log-linear models, in Proceedings of the 5th International Workshop on Semantic Evaluation (Uppsala: Association for Computational Linguistics), 264–267.

[B10] Chen Y.-C. Li L. Yu L. El Kholy A. Ahmed F. Gan Z.. (2020). Uniter: universal image-text representation learning, in European Conference on Computer Vision (Glasgow: Springer), 104–120.

[B11] Chu C. Wang R. (2018). A survey of domain adaptation for neural machine translation. CoRR, abs/1806.00258.

[B12] Costa A. D. (2020). A traduo por mquina enriquecida semanticamente com frames e paps qualia (Ph.D. thesis), Universidade Federal de Juiz de Fora, Juiz de Fora, Brazil.

[B13] Costa A. D. Marim M. C. Matos E. E. S. Torrent T. T. (2022). Domain adaptation in neural machine translation using a qualia-enriched framenet. arXiv [Preprint]. arXiv: 2202.10287.

[B14] Croft W. (1993). The role of domains in the interpretation of metaphors and metonymies. Cogn. Linguist. 4, 335–370.

[B15] Czulo O. Ziem A. Torrent T. T. (2020). Beyond lexical semantics: notes on pragmatic frames, in Proceedings of the International FrameNet Workshop 2020: Towards a Global, Multilingual FrameNet (Marseille: European Language Resources Association), 1–7.

[B16] de Marneffe M. C. Manning C. D. Nivre J. Zeman D. (2021). Universal dependencies. Comput. Linguist. 47, 255–308. 10.1162/coli_a_00402

[B17] Devlin J. Chang M.-W. Lee K. Toutanova K. (2019). BERT: pre-training of deep bidirectional transformers for language understanding, in Proceedings of the 2019 Conference of the North American Chapter of the Association for Computational Linguistics: Human Language Technologies, Volume 1 (Long and Short Papers) (Minneapolis, MN: Association for Computational Linguistics), 4171–4186.

[B18] Diederich J. (1990). Spreading activation and connectionist models for natural language processing. Theor. Linguist. 16, 25–64. 24324234

[B19] Dolbey A. Ellsworth M. Scheffczyk J. (2006). Bioframenet: a domain-specific framenet extension with links to biomedical ontologies, in KR-MED (Baltimore, MD).

[B20] Elliott D. (2018). Adversarial evaluation of multimodal machine translation, in Proceedings of the 2018 Conference on Empirical Methods in Natural Language Processing (Brussels), 2974–2978.

[B21] Elliott D. Frank S. Barrault L. Bougares F. Specia L. (2017). Findings of the second shared task on multimodal machine translation and multilingual image description, in Proceedings of the Second Conference on Machine Translation (Copenhagen: Association for Computational Linguistics), 215–233. 10.18653/v1/W17-4718

[B22] Elliott D. Frank S. Simaan K. Specia L. (2016). Multi30K: Multilingual English-German Image Descriptions, in Proceedings of the 5th Workshop on Vision and Language (Berlin: Association for Computational Linguistics), 70–74. 10.18653/v1/W16-3210

[B23] Fillmore C. J. (1977). The case for case reopened, in Syntax and Semantics, Vol. 8: Grammatical Relations, eds P. Cole, and J. M. Sadock (New York, NY: Academic Press), 59–81.

[B24] Fillmore C. J. (1982). Frame Semantics, in Linguistics in the Morning Calm, ed The Linguistics Society of Korea (Seoul: Hanshin Publishing Co.), 111–138.

[B25] Fillmore C. J. (1985). Frames and the semantics of understanding. Quaderni di Semantica 6, 222–254.

[B26] Fillmore C. J. Baker C. (2009). A frames approach to semantic analysis, in The Oxford Handbook of Linguistic Analysis, eds B. Heine, and H. Narrog (Oxford: Oxford University Press), 791–816.

[B27] Fillmore C. J. Lee-Goldman R. Rhodes R. (2012). The framenet constructicon, in Sign-Based Construction Grammar (Stanford, CA: CSLI), 309–372.

[B28] Fillmore C. J. Petruck M. R. Ruppenhofer J. Wright A. (2003). Framenet in action: the case of attaching. Int. J. Lexicography 16, 297–332. 30322234

[B29] Gamonal M. A. (2017). Modelagem lingu-stico-computacional de meton-mias na base de conhecimento multil-ngue (m.knob) da FrameNet Brasil (Ph.D. thesis), Federal University of Juiz de Fora, Juiz de Fora, Brazil.

[B30] Gangemi A. (2010). What's in a schema? in Ontology and the Lexicon: A Natural Language Processing Perspective, eds C. Huang, N. Calzolari, A. Gangemi A. Lenci, A. Oltramari, and L. Prevot (Cambridge: Cambridge University Press), 144–182. 10.1017/CBO9780511676536.010

[B31] Gruber T. R. (1995). Toward principles for the design of ontologies used for knowledge sharing? Int. J. Hum. Comput. Stud. 43, 907–928. 10.1006/ijhc.1995.1081

[B32] Hodosh M. Young P. Hockenmaier J. (2013). Framing image description as a ranking task: data, models and evaluation metrics. J. Artif. Intell. Res. 47, 853–899.

[B33] Huang C.-R. Calzolari N. Gangemi A. Lenci A. Oltramari A. Prévot L. (2010). Ontology and the Lexicon. Cambridge, MA: Cambridge University Press.

[B34] Khayrallah H. Thompson B. Duh K. Koehn P. (2018). Regularized training objective for continued training for domain adaptation in neural machine translation, in Proceedings of the 2nd Workshop on Neural Machine Translation and Generation (Melbourne, VIC: Association for Computational Linguistics), 36–44.

[B35] Kuznetsova A. Rom H. Alldrin N. Uijlings J. Krasin I. Pont-Tuset J.. (2018). The open images dataset v4: Unified image classification, object detection, and visual relationship detection at scale. arXiv [Preprint]. arXiv:1811.00982.

[B36] Lakoff G. (1979). The contemporary theory of metaphor, in Metaphor and Thought, eds A. Ortony (Cambridge: Cambridge University Press), 202–251.

[B37] Lakoff G. (1987). Women, Fire, and Dangerous Things: What Categories Reveal About the Mind. Chicago: University of Chicago Press.

[B38] Lakoff G. Turner M. (1989). More Than Cool Reason: A Field Guide To Poetic Metaphor. Chicago: The University of Chicago Press.

[B39] Lala C. Specia L. (2018). Multimodal lexical translation, in Proceedings of the Eleventh International Conference on Language Resources and Evaluation (LREC 2018) (Miyazaki).

[B40] Lenci A. (2001). Building an ontology for the lexicon: Semantic types and word meaning, in Ontology-Based Interpretation of Noun Phrases: Proceedings of the First International OntoQuery Workshop (Kolding).

[B41] Lenci A. Busa F. Ruimy N. Gola E. Monachini M. Calzolari N.. (2000). SIMPLE _ Linguistic Specifications. Pisa: University of Pisa and Institute of Computational Linguistics of CNR. 1–405.

[B42] Li L. H. Yatskar M. Yin D. Hsieh C.-J. Chang K.-W. (2019). Visualbert: A simple and performant baseline for vision and language. arXiv [Preprint]. arXiv:1908.03557.

[B43] Li X. Yin X. Li C. Zhang P. Hu X. Zhang L.. (2020a). Oscar: object-semantics aligned pre-training for vision-language tasks, in European Conference on Computer Vision (Glasgow: Springer), 121–137.

[B44] Li Z. Qu L. Haffari G. (2020b). Context dependent semantic parsing: A survey, in Proceedings of the 28th International Conference on Computational Linguistics (Barcelona: International Committee on Computational Linguistics), 2509–2521. 10.18653/v1/2020.coling-main.226

[B45] Lin T.-Y. Maire M. Belongie S. Hays J. Perona P. Ramanan D.. (2014). Microsoft coco: common objects in context, in European Conference on Computer Vision (Zurich: Springer), 740–755.

[B46] LoBue P. Yates A. (2011). Types of common-sense knowledge needed for recognizing textual entailment, in Proceedings of the 49th Annual Meeting of the Association for Computational Linguistics: Human Language Technologies (Portland, OR: Association for Computational Linguistics), 329–334.

[B47] Ohara K. (2018). The relations between frames and constructions: a proposal from the Japanese FrameNet Constructicon, in Constructicography: Constructicon Development Across Languages, eds B. Lyngfelt, L. Borin, K. Ohara, and T. T. Torrent (Amsterdam: John Benjamins), 141–164.

[B48] Papineni K. Roukos S. Ward T. Zhu W.-J. (2002). Bleu: a method for automatic evaluation of machine translation, in Proceedings of the 40th Annual Meeting of the Association for Computational Linguistics (Philadelphia, PA: Association for Computational Linguistics), 311–318.

[B49] Peters M. E. Neumann M. Iyyer M. Gardner M. Clark C. Lee K.. (2018). Deep contextualized word representations, in Proceedings of the 2018 Conference of the North American Chapter of the Association for Computational Linguistics: Human Language Technologies, Volume 1 (Long Papers) (New Orleans: Association for Computational Linguistics) 2227–2237.

[B50] Plummer B. A. Wang L. Cervantes C. M. Caicedo J. C. Hockenmaier J. Lazebnik S. (2015). Flickr30k entities: collecting region-to-phrase correspondences for richer image-to-sentence models, in Proceedings of the IEEE International Conference on Computer Vision (Santiago), 2641–2649.

[B51] Pustejovsky J. (1995). The Generative Lexicon. Cambridge, MA: MIT Press.

[B52] Qi D. Su L. Song J. Cui E. Bharti T. Sacheti A. (2020). Imagebert: Cross-modal pre-training with large-scale weak-supervised image-text data. arXiv [Preprint]. arXiv:2001.07966.

[B53] Radford A. Kim J. W. Hallacy C. Ramesh A. Goh G. Agarwal S.. (2021). Learning transferable visual models from natural language supervision. arXiv [Preprint]. arXiv:2103.00020. 33617450

[B54] Ringgaard M. Gupta R. Pereira F. C. (2017). Sling: A framework for frame semantic parsing. arXiv [Preprint]. arXiv:1710.07032.

[B55] Rogers A. Kovaleva O. Rumshisky A. (2020). A primer in BERTology: what we know about how BERT works. Trans. Assoc. Comput. Linguist. 8, 842–866. 10.1162/tacl_a_00349

[B56] Ruppenhofer J. Ellsworth M. Schwarzer-Petruck M. Johnson C. R. Baker C. F. Scheffczyk J. (2016). FrameNet II: Extended Theory and Practice. Berkeley, CA: International Computer Science Institute.

[B57] Sap M. Shwartz V. Bosselut A. Choi Y. Roth D. (2020). Commonsense reasoning for natural language processing, in Proceedings of the 58th Annual Meeting of the Association for Computational Linguistics: Tutorial Abstracts (Online: Association for Computational Linguistics), 27–33.

[B58] Scheffczyk J. Baker C. F. Narayanan S. (2010). Studies in natural language processing, in Reasoning over Natural Language Text by Means of FrameNet and Ontologies, Chapter 4 (Cambridge: Cambridge University Press), 53–71.

[B59] Schifrrin D. (1994). Approaches to Discourse. Cambridge, MA: Blackwell.

[B60] Sharma P. Ding N. Goodman S. Soricut R. (2018). Conceptual captions: a cleaned, hypernymed, image alt-text dataset for automatic image captioning, in Proceedings of the 56th Annual Meeting of the Association for Computational Linguistics (Volume 1: Long Papers) (Melbourne, VIC), 2556–2565.

[B61] Smith N. A. (2020). Contextual Word Representations: Putting Words into Computers. New York, NY: Association for Computing Machinery. 10.1145/3347145

[B62] Snover M. Dorr B. Schwartz R. Micciulla L. Makhoul J. (2006). A study of translation edit rate with targeted human annotation, in Proceedings of the 7th Conference of the Association for Machine Translation in the Americas: Technical Papers (Cambridge, MA: Association for Machine Translation in the Americas), 223–231.

[B63] Specia L. Frank S. SimaAn K. Elliott D. (2016). A shared task on multimodal machine translation and crosslingual image description, in Proceedings of the First Conference on Machine Translation: Volume 2, Shared Task Papers (Berlin), 543–553.

[B64] Su W. Zhu X. Cao Y. Li B. Lu L. Wei F.. (2019). Vl-bert: Pre-training of generic visual-linguistic representations. arXiv [Preprint]. arXiv:1908.08530.

[B65] Swayamdipta S. Thomson S. Dyer C. Smith N. A. (2017). Frame-semantic parsing with softmax-margin segmental rnns and a syntactic scaffold. arXiv [Preprint]. arXiv:1706.09528.

[B66] Tan H. Bansal M. (2019). LXMERT: Learning Cross-Modality Encoder Representations from Transformers, in Proceedings of the 2019 Conference on Empirical Methods in Natural Language Processing and the 9th International Joint Conference on Natural Language Processing (Hong Kong: Association for Computational Linguistics), 5100–5111. 10.18653/v1/D19-1514

[B67] Thompson B. Gwinnup J. Khayrallah H. Duh K. Koehn P. (2019). Overcoming catastrophic forgetting during domain adaptation of neural machine translation, in Proceedings of the 2019 Conference of the North American Chapter of the Association for Computational Linguistics: Human Language Technologies, Volume 1 (Long and Short Papers) (Minneapolis, MN: Association for Computational Linguistics), 2062–2068.

[B68] Torrent T. T. Ellsworth M. (2013). Behind the labels: criteria for defining analytical categories in framenet brasil. Veredas-Revista de Estudos Linguisticos 17, 44–66.

[B70] Torrent T. T. Matos E. E. Lage L. M. Laviola A. da Silva Tavares T. de Almeida V. G.. (2018). Towards continuity between the lexicon and the constructicon in FrameNet Brasil, in Constructicography: Constructicon Development Across Languages, eds B. Lyngfelt, L. Borin, K. Ohara, and T. T. Torrent (Amsterdam: John Benjamins), 107–140.

[B71] Tsatsaronis G. Vazirgiannis M. Androutsopoulos I. (2007). Word sense disambiguation with spreading activation networks generated from thesauri, in IJCAI, vol. 27 (Hyderabad), 223–252.

[B72] Vedantam R. Lawrence Zitnick C. Parikh D. (2015). Cider: consensus-based image description evaluation, in Proceedings of the IEEE Conference on Computer Vision and Pattern Recognition (Boston, MA), 4566–4575.

[B73] Wang Z. Yu J. Yu A. W. Dai Z. Tsvetkov Y. Cao Y. (2021). Simvlm: Simple visual language model pretraining with weak supervision. arXiv [preprint]. arXiv:2108.10904.

[B74] Xia P. Wu S. Van Durme B. (2020). Which *BERT? a survey organizing contextualized encoders, in Proceedings of the 2020 Conference on Empirical Methods in Natural Language Processing (EMNLP) (Online: Association for Computational Linguistics), 7516–7533.x

[B75] Yao S. Wan X. (2020). Multimodal transformer for multimodal machine translation, in Proceedings of the 58th Annual Meeting of the Association for Computational Linguistics (Online), 4346–4350.

[B76] Young P. Lai A. Hodosh M. Hockenmaier J. (2014). From image descriptions to visual denotations: new similarity metrics for semantic inference over event descriptions. Trans. Assoc. Comput. Linguist. 2, 67–78. 10.1162/tacl_a_00166

[B77] Zhang P. Li X. Hu X. Yang J. Zhang L. Wang L.. (2021). Vinvl: revisiting visual representations in vision-language models, in Proceedings of the IEEE/CVF Conference on Computer Vision and Pattern Recognition (Nashville, TN), 5579–5588.

[B78] Zhou H. Huang M. Zhu X. (2016). Context-aware natural language generation for spoken dialogue systems, in Proceedings of COLING 2016, the 26th International Conference on Computational Linguistics: Technical Papers (Osaka), 2032–2041.

